# Solution properties of spherical gold nanoparticles with grafted DNA chains from simulation and theory

**DOI:** 10.1039/d2na00377e

**Published:** 2022-08-19

**Authors:** Fernando Vargas-Lara, Francis W. Starr, Jack F. Douglas

**Affiliations:** Departments of Physics & Molecular Biology & Biochemistry, Wesleyan University Middletown CT 06459 USA lvargaslara@wesleyan.edu; Materials Science & Engineering Division, National Institute of Standards and Technology Gaithersburg Maryland 20899 USA jack.douglas@nist.gov

## Abstract

There has been a rapidly growing interest in the use of functionalized Au nanoparticles (NPs) as platforms in multiple applications in medicine and manufacturing. The sensing and targeting characteristics of these NPs, and the realization of precisely organized structures in manufacturing applications using such NPs, depend on the control of their surface functionalization. NP functionalization typically takes the form of polymer grafted layers, and a detailed knowledge of the chemical and structural properties of these layers is required to molecularly engineer the particle characteristics for specific applications. However, the prediction and experimental determination of these properties to enable the rational engineering of these particles is a persistent problem in the development of this class of materials. To address this situation, molecular dynamic simulations were performed based on a previously established coarse-grained single-stranded DNA (ssDNA) model to determine basic solution properties of model ssDNA-grafted NP-layers under a wide range of conditions. In particular, we emphasize the calculation of the hydrodynamic radius for ssDNA-grafted Au NPs as a function of structural parameters such as ssDNA length, NP core size, and surface coverage. We also numerically estimate the radius of gyration and the intrinsic viscosity of these NPs, which in combination with hydrodynamic radius estimates, provide valuable information about the fluctuating structure of the grafted polymer layers. We may then understand the origin of the commonly reported variation in effective NP “size” by different measurement methods, and then exploit this information in connection to material design and characterization in connection with the ever-growing number of applications utilizing polymer-grafted NPs.

## Abstract 2

Theoretically estimated change in hydrodynamic radius of a DNA nanoparticle *versus* the corresponding simulation estimate based on the program ZENO, in combination with molecular dynamics simulation to generate representative chain configurations.

## Graphical abstract

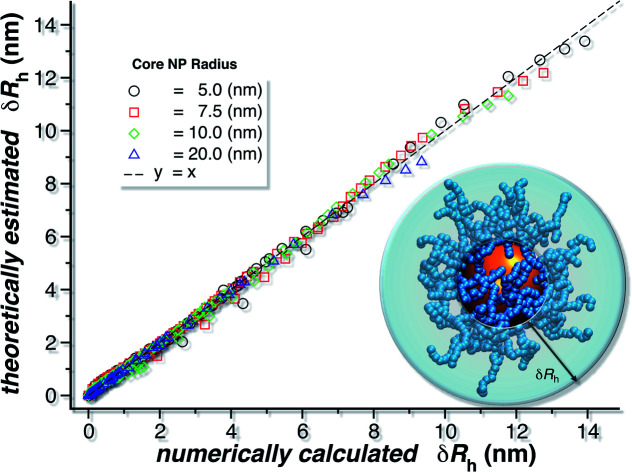

The nature of the surface functionalization of nanoparticles (NPs) is the critical determinant of their behavior. Control over this surface layer, and its response to environmental conditions, such as temperature and pH, enables a broad range of desirable characteristics to be programmed into NPs. The physical nature of the grafted polymer layer is thus a matter of paramount interest in the numerous applications of polymer-grafted NPs. Here, we computationally investigate spherical NPs with a grafted polymer layer with particular emphasis on Au NPs grafted with single stranded DNA (ssDNA) as a model system of this kind. In biophysical applications of this type, and similar polymer-grafted NPs, the presence of the polymer layer is crucial for the targeted delivery of the NPs to tumors and tissues and tissue engineering applications,^1–3^ many developing therapeutic applications for the diagnosis and treatment of disease,^4–9^ the detection of pathogenic viruses in the environment and the treatment of viral diseases^10^ and the detection of environmental heavy metals and pollutants in the water supply^11–13^ and nucleic acids^14^ based on calorimetric sensing.^15^ Notable applications of polymer grafted NPs are also under development in connection with gene delivery, bio-imaging, photothermal and photodynamic therapies^16–18^ and RNA grafted NPs have been found to be effective in modulating the gene expression of plants^19^ so there are emerging applications of nucleic acid grafted NPs in agriculture.^20^ In addition to this tremendous activity in the field of medical science, there have also been many materials science applications using these particles^21–27^ in which there are complementary strand–strand interactions that allow for formation of functional self-assembled nanostructures and even macroscopic crystals.^28,29^ In short, polymer grafted NPs are undergoing an explosion of activity in many fields of science and technology.

While these manifold applications are scientifically interesting, and practically of great importance, the present paper is focused on understanding the conformational structure of the grafted ssDNA chains, and the collective properties of these grafted layers and the NP as a whole, as evidenced by static properties such the radius of gyration (*R*_g_) of the polymer grafted NPs and hydrodynamic properties such as the hydrodynamic radius (*R*_h_) and intrinsic viscosity ([*η*]). Qualitatively, the solution properties of polymer grafted NPs have much in common with star polymers in solution,^30^ which is natural since polymer grafted NPs in the limit that the particle core particle has a size comparable to the polymer statistical segment become equivalent to star polymers. We note that the magnitude of *R*_h_ has emerged as one of the best correlates with biophysically relevant physicochemical properties of polymer grafted Au NPs.^31^

Polymer grafted NPs also bear some relation to “microgel” particles formed by polymerizing microemulsion droplets,^32–36^ where the nearly spherical particles formed under moderate cross-linking density are often found to have a relatively dense core and diffuse periphery whose dimensions has a size on the same order of magnitude as the core region of these particles. A striking signature of NPs and polymers of this type is that the ratio of *R*_h_/*R*_g_ can be much larger than the value of this ratio for hard spheres, 
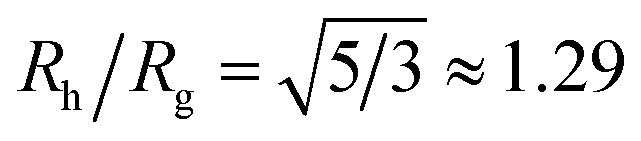
. Since the size of the solid cores of polymer grafted NPs are often larger or comparable to the thickness of the grafted layer and the grafting density of layers grafted from solution is normally moderate, polymer grafted NPs also tend to exhibit *R*_h_/*R*_g_ values significantly larger than the hard sphere value. This trend of exceeding the hard sphere value is also predicted for dendrimer polymers at high generation number^37,38^ and we may expect this general tendency in hyperbranched polymeric materials. Values of *R*_h_/*R*_g_ significantly greater than hard sphere value have also been observed in the molten globular state of collapsed polymer chains in solution^39,40^ and this phenomenon has also been observed in complexes polyethyleneamine and DNA of interested in drug delivery applications.^41^ The common thread in all these systems is occurrence of compact, nearly spherical particles having a dense core and diffuse outer periphery, a basic physical characteristic of polymer-grafted NPs.

Part of the motivation of the present study has come from the fact that many previous studies of polymer chains grafted on NPs and other surfaces have simply assumed that the chains adopt an extended conformation in which the grafted polymer chains take the form of polymer “brushes” in which the polymer chains are often imagined to adopt rod-like configurations oriented roughly normal to the surface to which they are grafted.^42^ Our simulations clearly show that the polymer chain conformations instead are more similar to free chains in solution for the grafting densities normally encountered in applications. Our computational method also enables us to extract other additional basic characterization information about these NPs, such as their *R*_h_, *R*_g_, and particle shape fluctuations quantified by the variance of these solution properties. We first describe the molecular model of the DNA grafted NP, and then the computational approach utilized to calculate the solution properties of these NPs and the conformational properties of the grafted polymer chains and the grafted layer as function of molecular parameters.

## 1 Model of single-stranded DNA grafted nanoparticles

We next describe our computational approach to simulate and characterize ssDNA-grafted NPs. We first utilize molecular dynamics simulations (MD) to generate ssDNA-decorated AuNPs configurations and then we use path-integral calculations^43^ to determine *R*_h_ for the configurations obtained from MD. This approach has been successfully applied to the study of more complex DNA-based structures.^44^

**Fig. 1:**
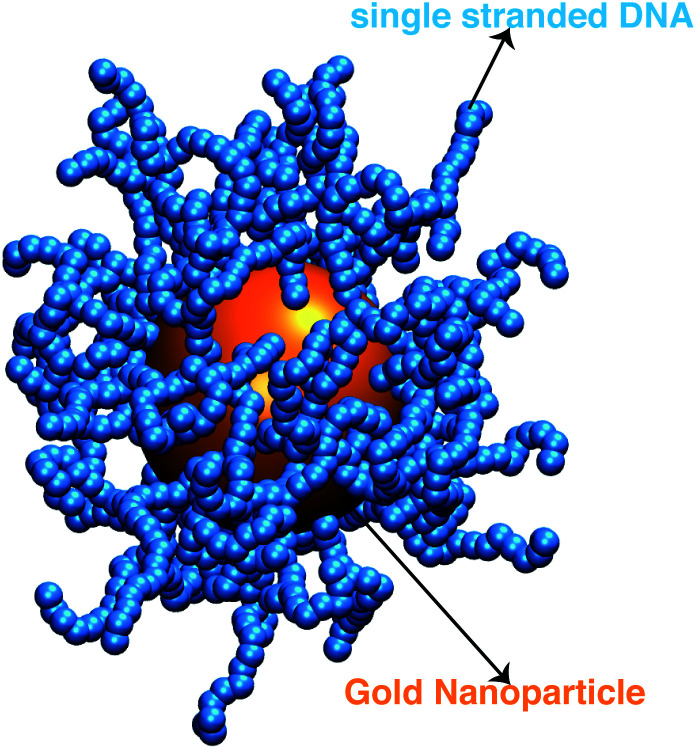
A representative configuration of a simulated ssDNA-grafted NP where the gold NP core is 5.0 nm in radius (orange sphere) and there are 60 ssDNA chains (connected blue spheres) grafted onto the NP core. Each ssDNA chain is formed by 18 **T** bases with *l*_p_ = 2.0 nm.

We model each ssDNA chain as a set of “beads” (blue spheres on Fig. 1) connected by “springs”.^45^ One end of each chain is tethered to a spherical symmetric particle (orange sphere on Fig. 1(a)) representing a gold core NP in the experimental system). The Weeks–Chandler–Andersen potential (*U*_WCA_) to simulate the soft core excluded volume interaction among all the beads and between each bead and the NP core,1
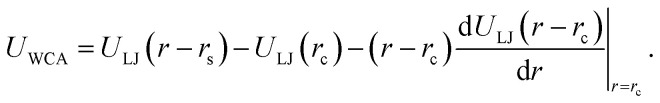
Here, *U*_LJ_ is the Lennard-Jones potential having *ε*_LJ_ and *σ* as energy and length parameters, respectively. Below, we define our energy units by the definition, *ε*_LJ_ = 1. The distance between two beads or one bead and the NP core is given by *r* and *r*_s_ is the distance that the origin of the potential has been shifted, and *r*_c_ is a cutoff distance. We chose *r*_c_ = *r*_s_ + 2^1/6^*σ* or *r*_c_ = *r*_s_ + 2.5*σ* to consider non-attractive or attractive interactions, respectively, among the particles to generate particles having a radius 
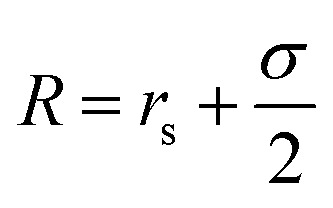
. For the blue beads we consider *r*_s_ = 0 and we set *σ* = 0.65 nm (ref. 46) the diameter of the bead which is equal to the ssDNA base-to-base distance. This value is a representative one for the “effective” ssDNA chain diameter inferred from the translocation of ssDNA chains on solid-state nanopores.^47^ For the NP core, we vary from *r*_s_ = 0.675 nm to *r*_s_ = 4.675 nm corresponding to NPs having core radii from *R* = 1.0 nm to *R* = 5.0 nm. The beads along the chain and the first bead of each chain are taken to be connected by a finitely extensible nonlinear elastic (FENE) springs described by the potential,2
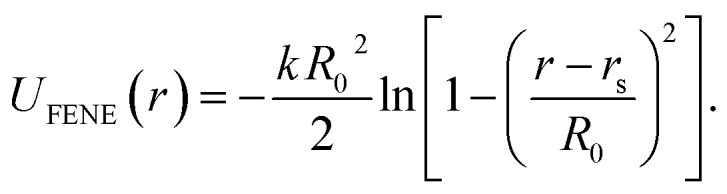


For this potential, we select *k* = 30/*σ*^2^ and *R*_0_ = 1.5*σ*. Additionally, we use a three-body angular potential (*U*_lin_(*θ*)) to control the chain stiffness,

3 *U*_lin_(*θ*) = *k*_lin_(1 + cos *θ*).

To model ssDNA, take *k*_lin_ = 1 to generate chains having persistence lengths *l*_p_ = (2.0 ± 0.1) nm, which is a representative value for ssDNA chains. This estimate is suitable under the salt concentrations, *c*_es_ ≥ 1 M NaCl,^48^ where charge interactions are largely screened. Here, *l*_p_ is defined as the characteristic length, where the bond orientation correlation function 
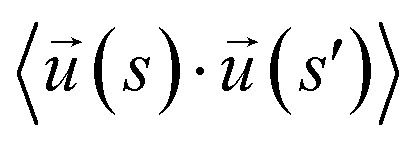
 reaches 1/*e*. Here, 
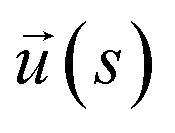
 is a unit vector tangent to the chain that is located at the position *s*. The ssDNA chains having chain lengths *L* = (5, 10, 18, or 40) Thymine bases (**T**), as in the experimental measurements. We vary the number of strands *N*_c_ attached to the NP core in the range 1 ≤ *N*_c_ ≤ 1000. An extension of this model to describe duplex DNA is discussed in ref. 49 and 50.

Our simulations utilize a canonical ensemble (*NVT*) with fixed reduced temperature, T* = 1.0*ε*/*k*_B_ for all our simulations (*k*_B_ is the Boltzmann constant) and it is controlled by using the Nosé–Hoover method.^51,52^ Our MD simulations were performed for ≥10^7^ time steps using the Large-scale Atomic/Molecular Massively Parallel Simulator (LAMMPS)^53^ and we render the left panel of Fig. 1 using the Visual Molecular Dynamics (VMD) program.^54^ We compute properties for 10^3^ different thermal equilibrated configurations (see Fig. 1(a)–(c)) using the path-integration program ZENO.^43^ These computations employ 10^5^ random walk trajectories to achieve low uncertainty. We determine the average value and standard deviation *σ*_s_ for each property and uncertainty quantification is provided by ZENO and indicated by error bars in plots when the values are larger than the point size. The quality of fitting the simulation results to functional forms will be provided by its correlation coefficient value, *ρ*.

## 2 Numerical results for the hydrodynamic radius of single-stranded DNA grafted nanoparticles

In a previous paper,^44^ we utilized the present model to calculating *R*_h_ of DNA grafted onto Au NPs having prescribed DNA chain lengths and grafting densities and variable size of the Au core of the NPs. The grafting density in this work was estimated following a methodology of Mirkin and coworkers,^55,56^ in which the DNA chains were grafted onto the Au NPs in solution up to a point of “saturation” conditions, at which no further DNA chains could be grafted. Our direct comparison of our simulations to the observed values of *R*_h_ with the grafting density and the size of the bare NPs independently determined yielded good agreement in this initial study, and we also modeled how these NPs altered the dimensions of DNA origami sheets to which the DNA grafted NPs were attached.

The extension of this work to describe the overage coverage of DNA on Au NPs independently requires a precise assay for estimating the number of grafted polymer chains on the NP surface, and corresponding dynamic light scattering measurements of DNA grafted NPs having a prescribed grafting density. Such well-characterized NPs and measurements are currently not available, and it is hoped that the present analysis stimulates an effort to synthesize and characterize such NPs. The methods described in the present study should also be helpful in the characterization of numerous other grafted NP systems that are currently being used in material science and medical applications.

**Fig. 2:**
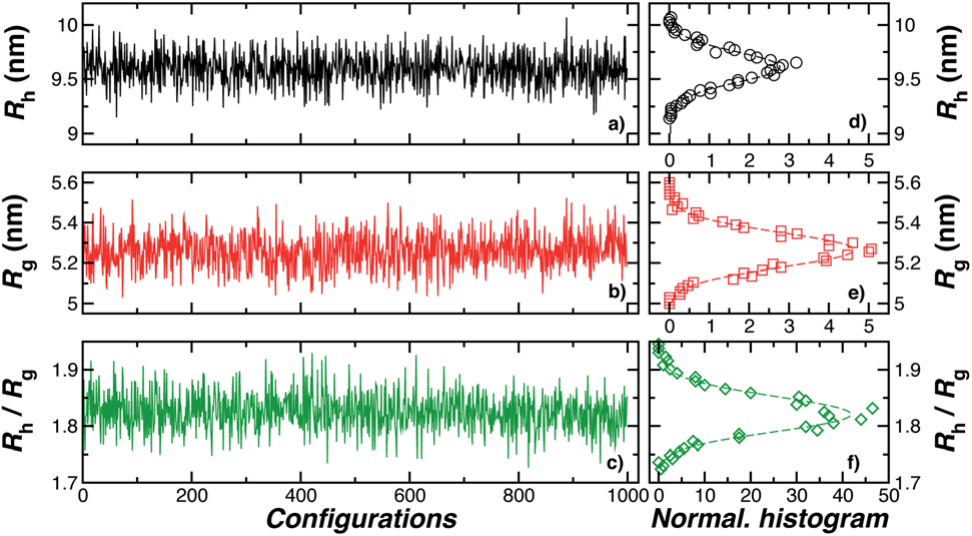
In panels (a), (b), and (c), the hydrodynamic radius *R*_h_, radius of gyration calculations *R*_g_, and the ratio *R*_h_/*R*_g_, respectively, for 10^3^ configurations similar to the one shown in Fig. 1. Panels (d), (e), and (f) show the normalized histogram for the properties calculated in (a), (b), and (c) from the ensemble of configurations. Dashed lines in panels (d), (e), and (f) are a guide to the eye.

We take advantage of our modeling to simulate polymer grafted NPs having grafting densities that are much higher than the “saturation grafting density” achieved when the polymer chains are grafted onto the bare metal NP from solution.^55,56^ The grafted density must ultimately be limited by the formation of a “brush-like” layer having a nearly uniform polymer segmental density, in which case the polymer-grafted NP becomes effectively similar to a hard sphere. This limiting hard sphere behavior can be achieved in the microemulsion derived NPs mentioned above in the limit of high grafting density^33–36^ or in polymer grafted NPs when the grafted polymer chains are grown directly from the surface of the NPs.^57,58^ However, the physical nature of the grafted polymer layer formed by grafting the polymer chains onto the NPs from solution has a rather different structure in which both the grafted ssDNA chains, and the grafted layer of the NP as a whole, exhibit appreciable conformational fluctuations (see snapshots of the simulated polymer grafted NPs in the inset of Fig. 2(a)). Thus, *R*_h_ of even a spherical NP having a fixed number of grafted polymer chains having a prescribed oligomer length will exhibit fluctuations that progressively diminish in magnitude as the polymer grafting density is progressively increased. Measurements of the fluctuations in *R*_h_ then offer some insight into the physical nature of the polymer grafted layer beyond a measure of the average thickness of the grafted layer. The fluctuations in the grafted layer shape also have independent interest for understanding the interaction between NPs in the presence of a fluctuating layer of grafted chains on NPs that can lead to self-assembly of the NPs into large scale string and sheet NP clusters in solution and in polymer nanocomposites.^59,60^ We address this fluctuation phenomenon in the next section.

As a final point relating to the range of application of our modeling, we note that progress has recently been made in creating DNA origami structures assemblies having diverse structural forms and in the modeling of these structures.^61–63^ The computational methodology based on numerical path integration (ZENO^43^) described in the present work should allow the facile computation of all these structures and a novel means to assess fluctuations in their geometry. Our emphasis on polymer grafted NPs is simply a particular model system from a much broader class of “nanoconstructs” that combine inorganic NPs and grafted chains involving nucleic acids such as DNA and RNA or polymers attached to functional drug molecules. The methods described in the present paper for characterizing ssDNA-grafted NPs have previously been successfully applied to duplex DNA solution properties over large mass range.^64,65^ We next focus on the solution properties of ssDNA-grafted Au NPs under physiologically relevant salt concentrations ≥0.1 M NaCl and pH 8.3,^48^ where electrostatic interactions can be reasonably neglected for the properties that we consider.

### 2.1 Size fluctuations for single-stranded DNA grafted nanoparticle

One of the basic physical features of polymer grafted NPs is that there size and shape fluctuates due the fluctuations of the individual flexible polymer chains and their collective fluctuations in the grafted polymer layer. These fluctuations have been observed to greatly influence the interparticle interactions in polymer grafted NPs in solution^66,67^ and polymer in polymer melts,^59^ and these fluctuations are no doubt germane to understanding their interactions with other molecules and to interfaces in a biophysical context. The extent of these fluctuations can be expected to depend on the length of the grafted chains, their stiffness, the grafting density on the NP, the polymer substrate interaction strength, *etc.* In the limit of very high grafting density, the so-called brush limit, we can expect these fluctuations to be small, the effect of polymer–surface interactions to be weak and the NP shape to be nearly spherical, but these fluctuations should be appreciable when the polymer chains are grafted onto the NP in solution, where interchain excluded volume interactions should limit the accessible grafting density.

In this section, we perform illustrative calculations of some basic solution properties that characterize NP, *R*_g_ and *R*_h_ for ssDNA-grafted NPs having a range of grafting densities where we calculate the distribution of these properties and the variances of their distribution to quantify the extent of fluctuations in these basic solution properties. We find that extent of fluctuations first grows monotonically until the point where a fully percolating layer is formed and the variance of these properties then progressively decreases, albeit slowly, with increasing grafting density. The peak in this distribution apparently denotes the onset of “saturation” in the coverage of the grafted chains.

**Fig. 3:**
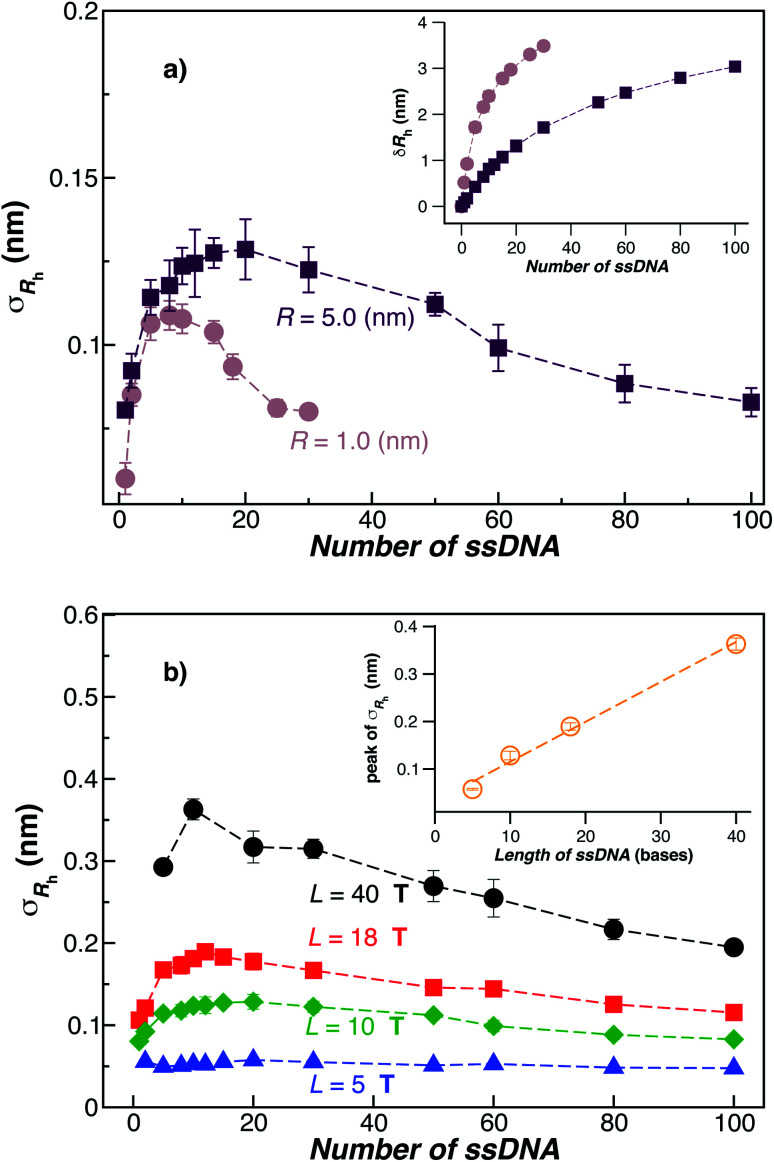
The standard deviation of the hydrodynamic radius *σ*_*R*_h__ as a function of the number of ssDNA chains attached to the NP core. (a) In this panel, we fix the ssDNA chain length *L* = 10 **T** and the ssDNA chain persistence length *l*_p_ = 2.0 nm and the core radius equals, *R* = (1.0 or 5) nm. The inset shows the difference *δR*_h_ = (*R*_h_ − *R*) for the same NPs. The dashed lines are guide the eyes. (b) The radius of the NP core *R* = 5.0 nm is fixed in this plot and the chain persistence length *l*_p_ = 2.0 nm and we vary the ssDNA chain length *L* = (5, 10, 18, or 40) **T** bases. Evidently, *σ*_*R*_h__ shows a peak value as the number of grafted chains is varied whose magnitude increases roughly linearly with *L* (see inset). Dashed lines are a guide to the eye.

Fig. 3(a) shows the standard deviation of the distribution of hydrodynamic radius *σ*_*R*_h__ as a function of the number of ssDNA chains *N* grafted to the NP, providing a quantification of the fluctuations in the shape of grafted NPs. Correspondingly, we see that *σ*_*R*_h__ estimated from simulation exhibits a peak value, presumably reflecting the formation of a percolating grafted layer, and beyond this point *σ*_*R*_h__ decreases progressively with increasing grafting densities, ultimately approaching the brush limit where *σ*_*R*_h__ = 0. The experimental values of *σ*_*R*_h__ beyond the saturation condition are roughly constant, although this quantity does exhibit significant fluctuations in the relatively high grafting density regime. We take the absence of a peak in *σ*_*R*_h__ and the rough constant magnitude of *σ*_*R*_h__ for large *N* as providing further evidence that the number of chains grafted to the Au NPs saturates for *σ*_*R*_h__ = 30 when *R* = 5.0 nm.

The peaking of *σ*_*R*_h__ in Fig. 3(a) with the number of grafted DNA chains depends on the NP core radius (*R*), the length of the ssDNA chains (*L*), as in shown in Fig. 3(b). The *σ*_*R*_h__ peak height is higher for a larger NP core and for larger ssDNA chains. Correspondingly, the number of grafted DNA chains at which *σ*_*R*_h__ peaks depends on the NP core radius *R*, an effect that evidently derives from the higher interfacial area of the larger NP. Similar trends are observed for the *σ*_*R*_g__ of these ssDNA-grafted NPs (results not shown). We then see that it is possible to “tune” the NP shape and mobility fluctuations by varying the NP core size, polymer chain length and chain grafting density, which in turn influences the mutual interaction of these NPs and their interaction with other molecules and surfaces. It is our impression from limited data that the grafting saturation condition occurs near the conditions in which *σ*_*R*_h__ peaks, corresponding to the formation of a “percolated” grafted layer in which the grafted chains start to come into contact and start to interact intermolecularly. However, this inference requires checking by further measurements in which is the polymer grafting density is better quantified. As noted before, this percolation transition has been directly imaged and modeled in the case of polymer chains grafted onto planar interfaces,^68^ and the sluggish chain dynamics associated with polymer topological interactions in adsorbing and desorbing polymer layers has also been investigated.^69,70^

We note that recent studies^71–73^ of the interaction of single stranded DNA polymer chains with Au NPs and planar Au surfaces without grafted DNA layers have indicated that this interaction is “exquisitely controlled by many factors including intermolecular forces, along with DNA composition and sequence”^71^ and the interaction is also strongly dependent on NP size, but it is not due to charge screening effects.^73^ The control of these interactions is important in many applications utilizing DNA grafted or adsorbed to Au NPs or planar surfaces and Koo *et al.*^71^ have reviewed this important topic with some of these applications in view. In our coarse-grained model of DNA grafted NPs, we model both excluded volume and polymer–substrate interactions by Lennard-Jones type interactions having a variable well-depth parameter describing the relative depth of the polymer–polymer and polymer–substrate attraction found in any particular system. This approach should apply well in cases where the interactions involved have a relative short-range interaction on the order of the segments of the polymer chains. While we expect this type of modeling to hold very well, we must admit that this type of modeling does not allow for an *a priori* prediction of the strength of the polymer surface interaction strength. This interaction parameter requires measurement for its determination under any solution condition of particular interest for a specific application. We note that the strong sensitivity of this interaction to NP size is a bit of a complication^73^ and this important effect is also prevalent in the context of the formation of bound protein layers on Au and other NPs. Lacerda *et al.*^74^ discuss this effect in some detail in the important case of the binding of representative blood proteins to Au NPS having a range of sizes where the size effect on the NP–protein interaction strength is quite apparent.

We suggest that it should be possible to gain further insights into this problem by studying the dimensions of grafted DNA layers by neutron reflectivity, as considered previously for synthetic polymers grafted to polymer layers,^75^ and by complementary molecular dynamics simulations.^76^ Recently, it has been shown that DNA grafted layers of high uniformity on Au surfaces can be synthesized,^77^ which could be used as the basis of this type of measurement. Neutron reflectivity studies on synthetic grafted layers have also shown that the polymer–substrate interaction can be controlled, and the dimensions of the grafted layer can controlled by “back-filling” the regions of the grafting substrate by very low mas polymers with end-groups that tune the polymer–surface interaction.^78^ This approach should be helpful in engineering of the polymer–surface interaction for numerous applications using DNA and other surface-grafted polymers bound to either through chemical bonds or physical association to substrates. We note that this approach can also be used to control the binding of polymers with grafted polymer layers onto interfaces.^79^

**Fig. 4:**
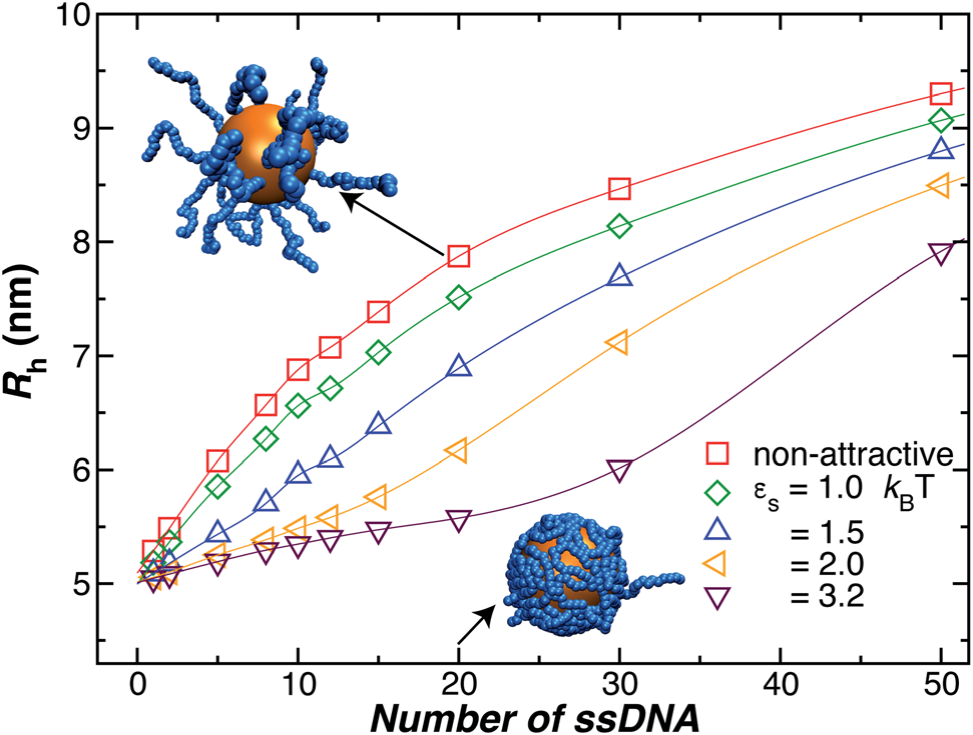
Influence of the polymer–nanoparticle interaction strength on the hydrodynamic radius *R*_h_ for a 5.0 nm NP as a function of the number of grafted strands, where each strand is formed by 18 **T** bases. We vary the interaction strength between the NP core and the ssDNA bases by modifying *ε*_s_, as it is shown in the legend. We see that attractive interactions can lead to an appreciable change in the thickness of the grafted polymer layer, the effect is especially significant at lower grafting densities. Lines guide the eye.

As a final remark, we emphasize that very few modeling studies of polymer-grafted NPs even consider the existence of a variable polymer–surface interaction strength in layers of polymers grafted to NPs and planar surfaces. Apparently, this approach to modeling these polymeric structures reflects a presumption that grafted polymer layers have a “brush-like” structure in which there is little opportunity to for the chain segments to access the boundary region so that the chains have little capacity to respond to this interaction by changing the conformational structure of the grafted layer. One of our main points in showing the data in Fig. 4 is to illustrate that the size of the NPs with grafted polymer layers having a grafting density and chain lengths representative of those utilized in measurements and applications of DNA grafted NPs depend sensitively on the NP–polymer interaction. This is just one of the implications of the deviation of real grafted layers from the idealized brush model often assumed in modeling polymer grafted NPs and interfaces. This sensitivity of the NP to the actuation of changes in the dimensions of the grafted layer by introducing molecules that segregate to the grafting surface, thereby altering the polymer–substrate interaction strength,^78^ implies that other molecules in solution (“impurities”) could affect the performance of these NPs in applications.

### 2.2 Influence of single-stranded DNA–nanoparticle core interaction

Another factor that may affect the configuration of the grafted chain layer is the influence of any attractive interaction between the surface of the Au NP core and the polymer grafted to the NP on the conformation of the polymer in the grafting polymer layer. Such interactions are normally neglected in ideal polymer “brush” layers, but, as we shall see, these layers are normally not “brush-like”, and it is important to assess the effect of the polymer–NP interactions on the size of grafted NPs.

In Fig. 4, we show the influence of the strength *ε*_s_ of an attractive interaction between the NP core and the grafted chains on *R*_h_. We see that this attractive interaction between the grafted polymer layer and the NP surface can evidently lead to an appreciable change in the thickness of the grafted polymer layer and the size of the NP, an effect that is especially large at lower grafting densities. When we compare the curves to our measurements for a 5 nm Au NP, we infer these interactions must be weak for our 5 nm radius NP. However, previous work has shown that the attractive interactions become stronger for blood proteins and much larger Au NPs,^74^ and we inferred a similar effect in our earlier work in Au NPs with grafted DNA.^44^ While the polymer–surface interaction does not appear to be a significant effect in our measurements on NPs as small as those considered in Fig. 4, we need to keep in mind that these interactions can be highly relevant to the thickness of grafted polymer layers in general.^71,80^

### 2.3 Influence of single-stranded DNA length

**Fig. 5:**
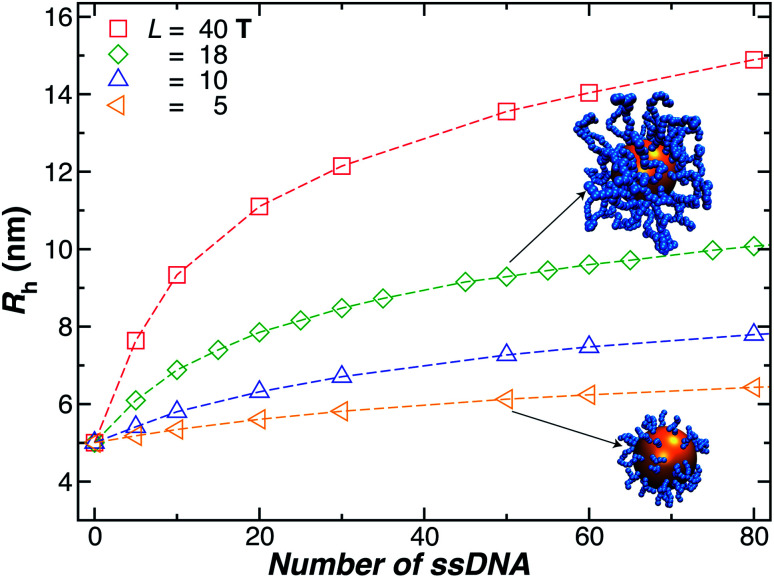
Nanoparticle hydrodynamic radius, *R*_h_, as a function of the number of ssDNA chains attached to the NP core with radius *r* = 5.0 nm. We vary the length of the chain *L*, as indicated in the legend. The open symbols are simulation results and the dashed lines guide the eye.

We next present our simulation results for NP decorated strands having different length, *L* = (5, 10, 18, or 40) **T** bases and different number of strands *N* in Fig. 5. We start by fixing the NP core size *R* = 5.0 nm and varying the number of ssDNA attached to the NP core. As expected, we find the change in *R*_h_ with increasing *N* is higher for larger chains.

**Fig. 6:**
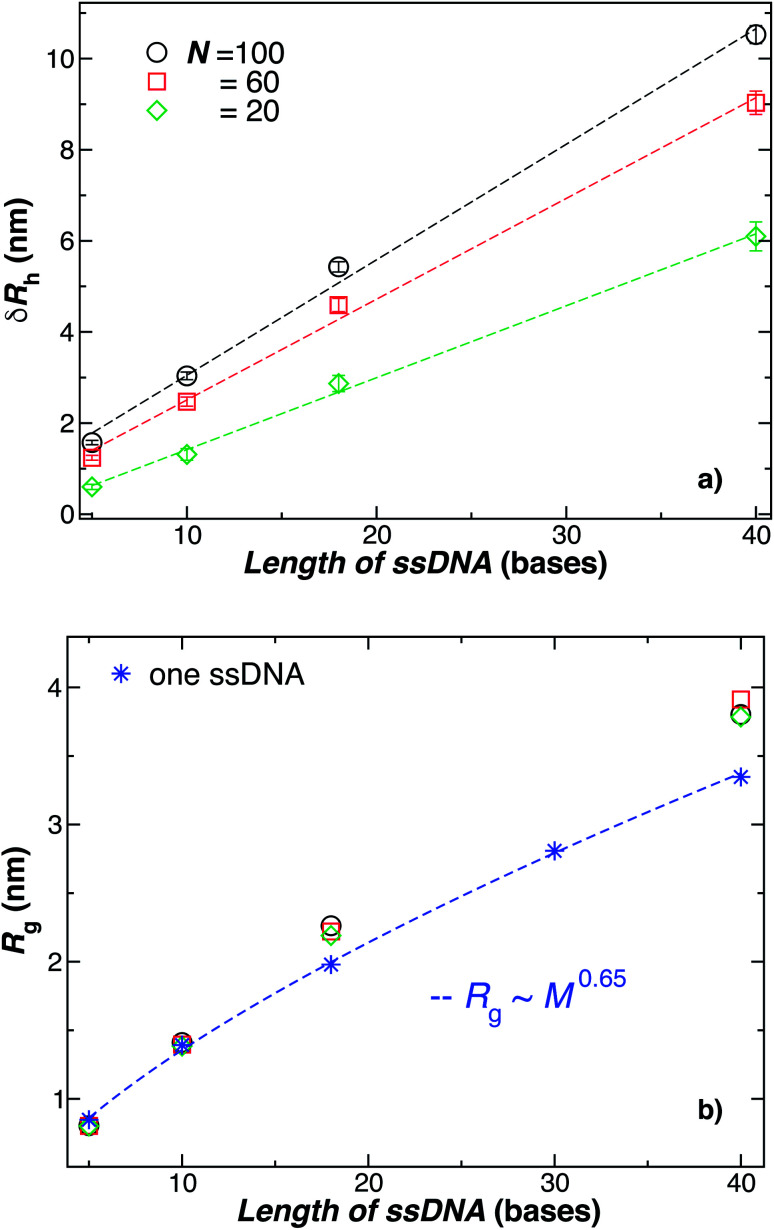
Comparison of the change in the NP radius from the presence of grafted polymer layer and the size of an individual polymer in solution. (a) The change in the hydrodynamic radius, *δR*_h_ = *R*_h_ − *R*, for a 5.0 nm in radius NP having grafted with 20, 60, or 100 ssDNA chains for a range of the ssDNA chain lengths, where the ssDNA chains have 5, 10, 18, or 40 **T** bases. The symbols are the data, and the dashed lines are linear regressions to the data with correlation coefficients, *ρ* ≥ 0.9. The nearly linear scaling of *δR*_h_ can give the misleading impression that the ssDNA chains are adopting a rod-like configuration.^81^ (b) The radius of gyration *R*_g_ for chains grafted onto the same NPs as in panel (a) (black circles, red squares, green diamonds) in comparison to *R*_g_ for individual ssDNA chains in solution (blue stars). The blue dashed line is a fit to the blue stars using a power law relation from where we obtain the scaling exponent *ν*. Here, *ν* ≈ 0.65 ± 0.007 with *ρ* = 0.99. We again conclude that the ssDNA chains attached to the NP core have a configurational structure similar to ssDNA chains in solution.

The primary reported evidence that has been interpreted as implying a “rod-like” ssDNA chain conformations of the chains grafted on the NP surface has been surmised based upon measurements of the effective *R*_h_ (obtained from the Stokes–Einstein relation and diffusion coefficient estimates from dynamic light scattering) changes relative to *R*_h_ of the NP core. In particular, a linear scaling in *δR*_h_ = *R*_h_ − *R* with length of the grafted polymer has been interpreted as implying the polymer chains are standing up straight as in the bristle of a paint brush.^81^ We indeed find this linear scaling in Fig. 6(a) for our *R* = 5.0 nm NP having grafted ssDNA chains onto the surface of a 5.0 nm radius NP core, but this apparent linear scaling certainty does not imply that the chains have been highly stretched “bristle-like form” shown in many cartoons of polymer brush layers. Fig. 6 shows *R*_g_ of the *individual* chains in the grafted layer for *N* = 20, 60, and 100 indicate that *R*_g_ is relatively *unchanged* from its value in solution, where the *M* scaling of *R*_g_ in solution is consistent with a short flexible polymer, *R*_g_ ∼ *M*^0.65^. Interchain and self-excluded volume interactions are apparently not strong in these grafted chain layers so that the brush cartoon of highly extended polymer chains is the grafted layer is not supported by our simulation observations, despite the nearly linear scaling of *δR*_h_ in Fig. 6(a).^81^

### 2.4 Influence of nanoparticle core size

The increase of *R*_h_ arising from an increase of the NP core size is rather easy to understand and this case has been discussed in a previous paper in the limit where a “saturated” layer of DNA chains was grafted onto the Au NP surfaces.^44^ For a given grafting density, the intercept of a plot of *R*_h_*versus* NP core size is a reasonable estimate of the thickness of the interfacial layer. However, the treatment of the case where the surface grafting density is not in the saturated layer limit is complicated by the uncertainty in estimating the surface grafting density when the NPs become much larger than a scale on the order of a few nm. For larger particles, we expect the attractive interaction between the Au core and the ssDNA chains to rapidly form a saturation polymer layer on the NP surface, making experimental estimates of the polymer grafting density highly uncertain. The signature of this effect is that *R*_h_ of the polymer-grafted NP has a size consistent with a saturating coverage almost regardless of the estimation of the nominal number of grafted polymer chains based on the method described in Section 2.1. There is an evident need to improve experimental estimates of the polymer grafting densities to account for the significant roles of the polymer grafting densities to account for the significant roles of the polymer–NP interfacial interaction and the polymer–polymer excluded volume interaction in forming these layers. Hydrodynamic measurements offer some insight into this problem, as discussed in Section 5.

## 3 Theoretical model for the hydrodynamic radius of spherical nanoparticles with grafted particles and polymers

We next develop a general model for the dependence of *R*_h_ on chain length, core size, and chain grafting density that can be used in this and other contexts where such polymer-grafted NPs need to be characterized. In particular, we provide useful relationships for the estimation of *R*_h_ as a function of the ssDNA-grafted NPs structural parameters. In the next subsections, we derive a relationship that describes *R*_h_ for our model NPs that should be transferable to Au NPs having arbitrary grafting densities and chain lengths, as well as many other types of polymer-grafted NPs in which the shape of the particle core and the molecular nature of the grafted polymer layer is varied. This development occurs through a succession of models a series of models of increasing molecular faithfulness of the individual polymer chains grafted onto a spherical nanoparticle (NP) structure (spheres, ellipsoids, semi-flexible polymer chains with excluded volume interactions), and we then extended the model to describe collections of these coarse-grained model polymers using a combination of molecular dynamics simulations of the conformational structure of the grafted polymer layers and the path-integration program ZENO.^43^ The reason for this procedure is the extreme complexity of the hydrodynamic properties of such layers and this approach allows us to systematically build on known results, which progressively extend and then validate by simulation, ultimately arriving at description in which we can describe the hydrodynamic properties of polymer grafted NPs where the chain conformational structure is explicitly considered, with some confidence.

### 3.1 Spherical nanoparticle with a single small grafted sphere

It is well-known that *R*_h_ of a spherical particle equals its radius, *R*_h_ = *R*, and as a first step, we consider how *R*_h_ of a spherical particle changes due to the addition of another small spherical particle having a radius *r*. First, consider the simplest case where the spheres are tangential, so that the center-to-center distances is always *r* + *R* (see Fig. 8). To determine the change in the hydrodynamic radius, *δR*_h_ = *R*_h_ − *R*, of spherical particles due to the presence of the second one (binary system), we recall the hydrodynamic–electrostatic analogy proposed by Douglas and coworkers^37,43^ that states the *R*_h_ of an object is approximately equal to the capacity *C* of a conducting particle having the same shape,

4 *R*_h_ ≈ *C*,

where the approximation has been established to hold to an accuracy ≈1%. In this relation, the units of *C* are chosen so that 4π*ε*_0_ = 1 where *ε*_0_ is the permittivity of the medium in which the sphere is placed and the charge is taken to be unity so that the capacity of a sphere then equals its radius, *C* = *R*. More generally, *C* has units of length in three dimensions.

We can take advantage of the exact solution for the self-capacity of two-touching spheres having radius, *R* and *r*, respectively, given by Russell^82^ to estimate *R*_h_ for two touching spheres of generally different diameter,5
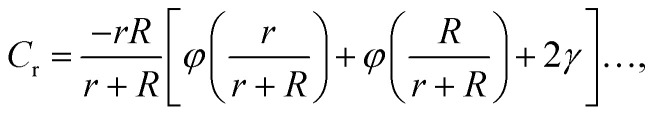
where *φ* is the logarithmic derivative of the gamma function and *γ* is the Euler constant, *γ* = 0.5772…. For the case when (*r*/*R*) < 1, *C*_r_ can be expanded in a power series in *r*/*R* (ref. 82) and we deduced a simple approximation for *δR*_h_, through resumation of the dominant terms in this expansion,6
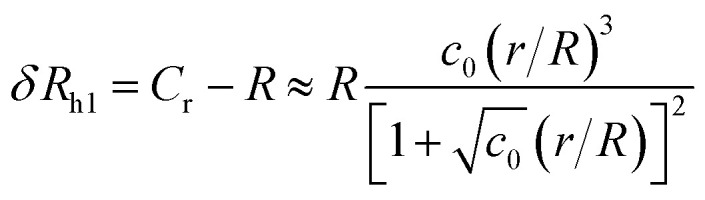


**Fig. 7:**
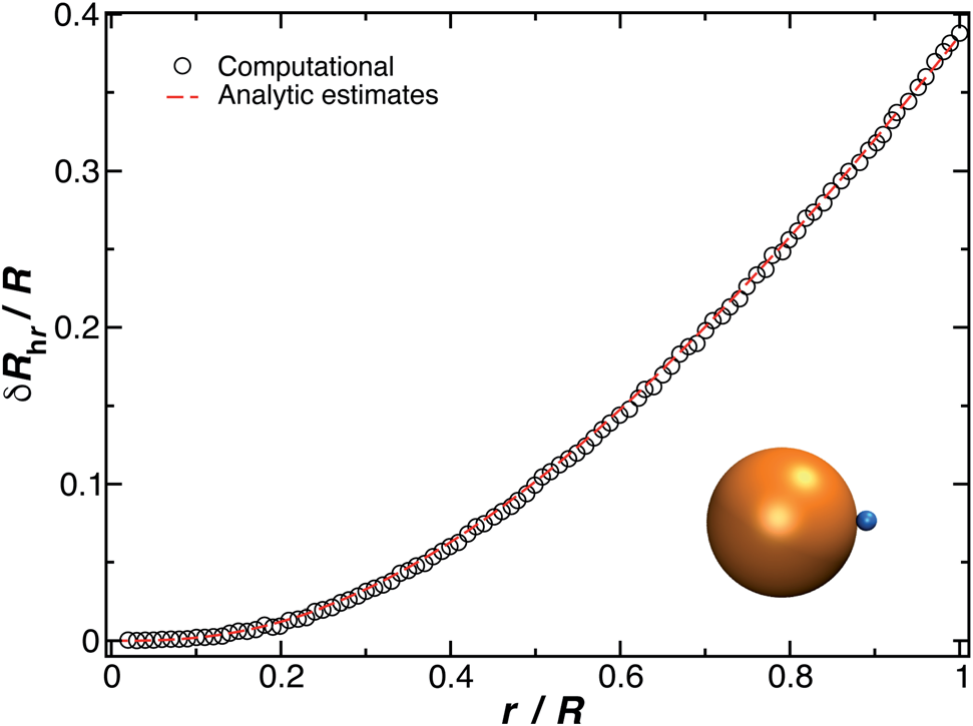
Normalized change in the hydrodynamic radius for a spherical particle (gold sphere with radius, *R*) with an attached small spherical particle (blue particle). The open symbols are calculations, and the dotted line is given by eqn (6) with *ρ* = 0.99.

The constant *c*_0_ is fixed by the exact result for *C*_r_ in the limit of *r*/*R* → 0 and *C*_r_(*r*/*R* = 1) to obtain an approximation for *C*_r_, and thus *δR*_h_ that cover a large range of *r*/*R*. In particular, solving the Laplace's equation with constant boundary conditions on the surface for two-tangential spheres having the same radii by consistency leads to precise prediction, *C*_r_ = 2 ln(2) = 1.386…*R*, so that *c*_0_ = 2.69678 (the best numerical estimate^83,84^ of *δR*_h1_ for two spheres having the same radius equals = 0.384*R*). Fig. 7 shows the results for *δR*_hr_/*R* as a function of the ratio *r*_h_/*R*, where *r*_h_ is the radius of the small sphere. The symbols were obtained by using the path-integration program ZENO, indicated as a line in eqn (6). No fitting parameters were required, demonstrating the predictive nature of eqn (6) over a large range of *r*/*R*.

### 3.2 Spherical nanoparticle with a grafted small ellipsoid

**Fig. 8:**
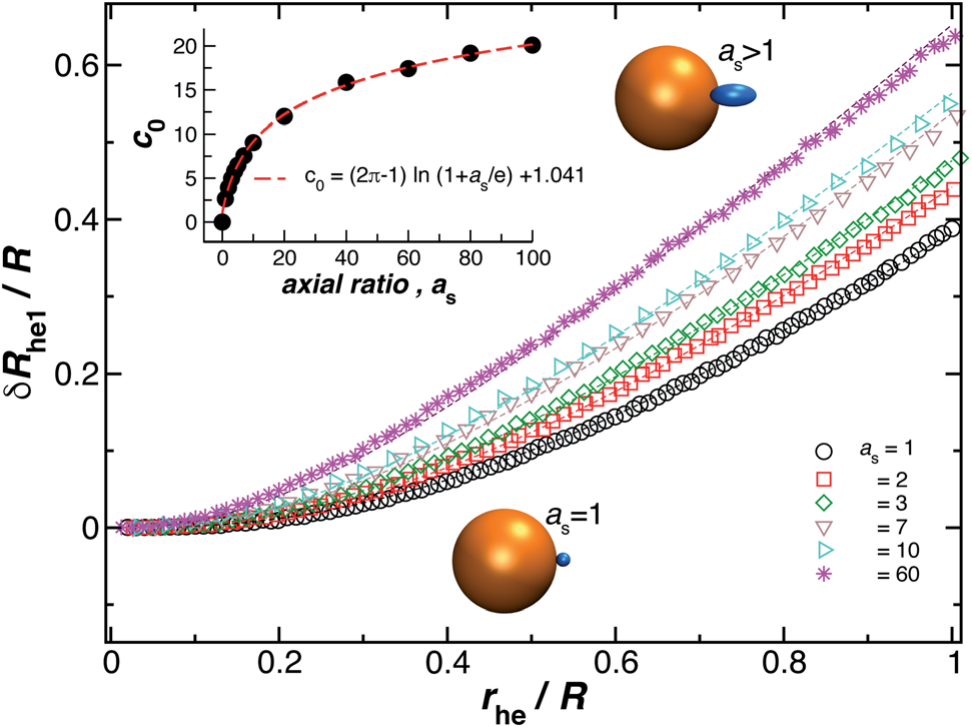
Normalized change in the hydrodynamic radius for a spherical particle (gold sphere) with an attached ellipsoidal particle (blue ellipsoid) having an axial ratio, *a*_s_. The open symbols are calculations and the dotted lines are fits to eqn (6), with correlation coefficients, *ρ* ≥ 0.96. From here we obtain the values of *c*_0_ and we plot them as a function of *a*_s_ in the figure inset. The red dotted line indicates a fit to eqn (6) with *ρ* ≥ 0.99.

We now turn our attention to the calculation of the change in *R*_h_ when an ellipsoidal particle rather than a sphere is attached to the surface of the relatively large sphere, *δR*_he_ (see Fig. 8). Here, the ellipsoid is defined by its three semiaxis *a*_1_, *a*_2_, *a*_3_ and for practical purpose, we only consider prolate ellipsoids, *a*_1_ = *a*_2_, < *a*_3_. For this case, we found the parameter *c*_0_ on eqn (6) depends on the ellipsoid axial ratio *a*_s_ = *a*_3_/*a*_1_. Fig. 8 shows the results for *δR*_he_ as a function of the ratio *r*_he_/*R*, where *r*_he_ is the *R*_h_ of the ellipsoid. Here, we vary the axial ratio as it is indicated in the legend of the main figure. The symbols were obtained by using the path-integration program ZENO and the lines are fits to eqn (6). We obtain the parameter *c*_0_ from the fits of the data to the eqn (6) and we find that it follows the empirical relationship extending the limiting spherical particle result in eqn (6),

7 *c*_0_ = (2π − 1)ln(1 + *a*_s_/*e*) + 1.041,

to a good approximation, and for the special case of spherical particles, *a*_s_ = 1, we get back *c*_0_ = 2.696. The inset on Fig. 8 shows *c*_0_ as a function of *a*_s_ as well as the fit to eqn (7) (red dashed line).

### 3.3 Spherical nanoparticle with a single-stranded DNA grafted chain

**Fig. 9:**
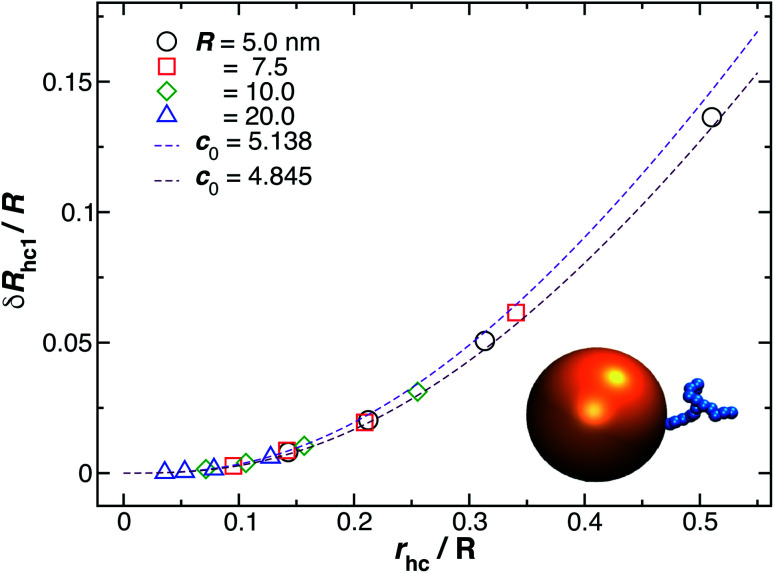
Normalized change in the hydrodynamic radius for a spherical particle (gold sphere) due to the addition of ssDNA chain (blue connected beads). Here, *r*_hc_ is the hydrodynamic radius of an isolated polymer chain in solution. The open symbols are calculations and the dotted lines are plots of eqn (6) considering two limit values of *c*_0_ described in the legend.

Fig. 9 shows the change in the *R*_h_ for a spherical particle (gold sphere) due to the addition of ssDNA chain (chain of blue beads in figure). The open symbols are calculations and the dotted lines are plots of eqn (6) considering two limiting values of *c*_0_. We now estimate *δR*_h1_, based on a model in which each ssDNA chain is oriented with the long axis defined by the long axis of *R*_g_ tensor of the polymer radius of gyration tensor is oriented perpendicular to the local attachment point on the sphere to which it is attached. The lengths of the principal axes of the radius of gyration tensor (*Λ*_1_, *Λ*_2_, *Λ*_3_) are obtained from the diagonalization of the radius of gyration tensor of the ssDNA in solution and the orientation of this effective ellipsoid is taken to be defined the long axis of this ellipsoid, which may be viewed a as coarse-grained model of the attached chain. Here we use the convention *Λ*_1_ ≤ *Λ*_2_ ≤ *Λ*_3_, and approximate the “axial ratio” as,8
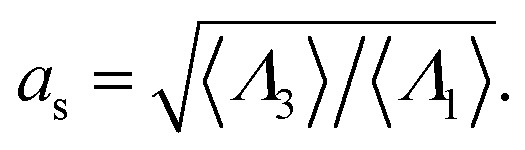


**Fig. 10:**
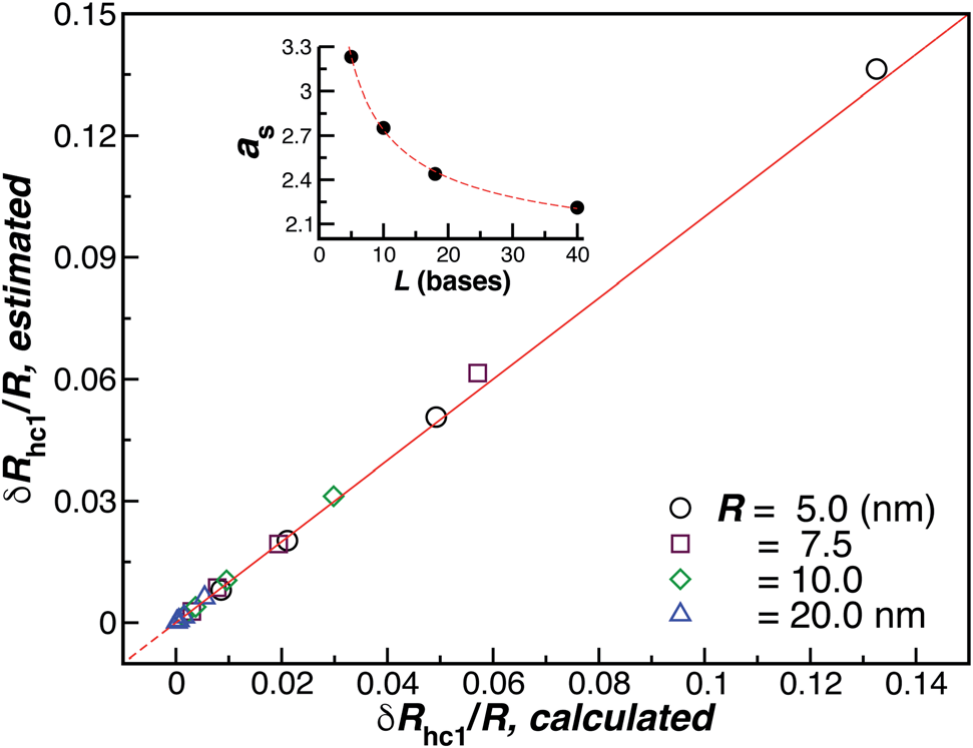
Analytic *versus* simulated estimates of *δR*_hc1_/*R* (ellipsoid approximation). The red line is a fit to the identity function *y* = *x*, with *ρ* = 0.99. The inset shows the axial, eqn (8), for the ssDNA chains in solution as a function of the chain length.

Fig. 10 shows a comparison between the estimated values for *δR*_h1_/*R* obtained by combining eqn (6) and (8) and the values calculated by using ZENO. We find that we can obtain a quite good approximation of *R*_h_ of a particle with a single grafted chain.

### 3.4 Spherical nanoparticle with many grafted small spheres and ellipsoids

We next graduate to the more physically interesting case of NPs having many attached particles or polymers. Building on the analysis before, we first consider *δR*_h_ for *N* small particles attached to the surface of the NP core particle. Within the electrostatic-hydrodynamic analogy indicated above, this problem is formally equivalent to the capacity of *N* capacitors on the surface of a sphere,^85^ which has been treated in previous studies of diffusion-limited reaction on the surface of cells. In particular, for a sphere with *N* randomly attached small spheres, we estimate *δR*_h_ by,^86–88^9
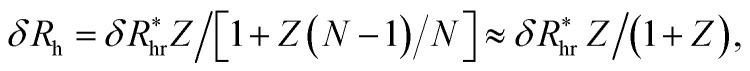
where 
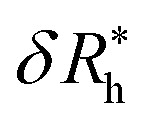
 is the saturation values of *δR*_h_, when the sphere is fully covered by a large (*N* ≫ 1) number *N* of small spheres, and 
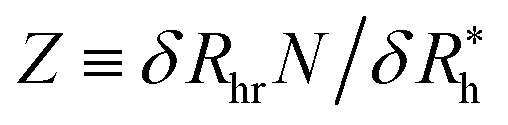
, where *δR*_rh_ is the single attached sphere property calculated above. We note that if *r* ≪ *R*, 
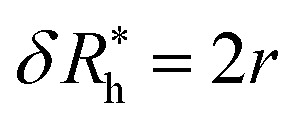
 and if *N* = 0, *δR*_h_ = 0.

Eqn (9) arises in the crossover description of the hydrodynamics of polymer chains and this functional function has been termed the “hydrodynamic penetration function”,^86^ a measure of the strength of the hydrodynamic interaction within the polymer coil. Here this term refers to the degree of hydrodynamic interaction strength within in the interfacial layer around the NP. The limit *Z* arrow infinity corresponds to the so-called “non-draining” limit where the layer is surface layer becomes a hydrodynamically impenetrable layer of uniform thickness. The crossover function in eqn (9) arises in many types of interacting polymer problems in addition to the hydrodynamic properties of polymers in solution–polymer excluded volume interactions, surface interacting polymers, capacity of complex conducting polymeric structures, *etc.*^86,89^

**Fig. 11:**
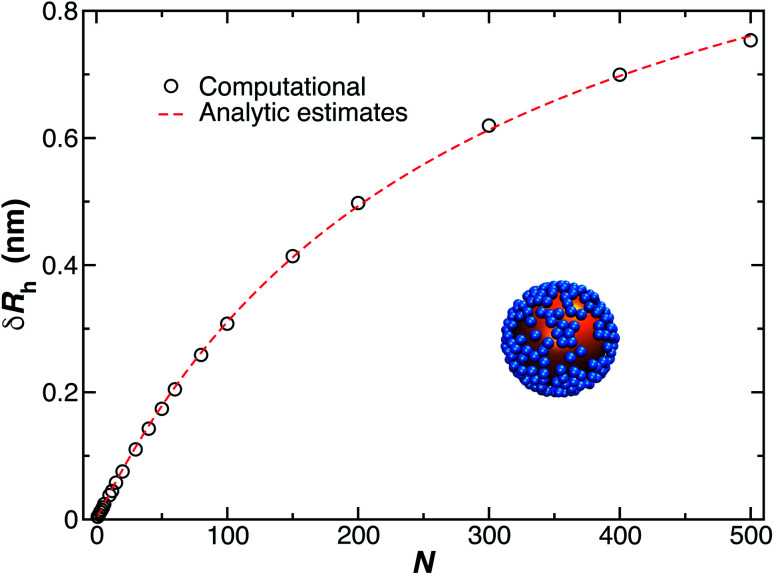
Change in the hydrodynamic radius for a spherical particle (gold sphere) due to the addition of *N* small spherical object (blue beads). We find the data is well described by eqn (9) (red dashed line, with *ρ* = 0.99).

Fig. 11 shows a comparison between the computational estimates (black circles) of *δR*_rh_ and the analytic estimates (red dashed line), eqn (9), where *C*_r_ and *R*_h_ are equated.

The treatment of many grafted ellipsoids or other objects of fixed and general shape to a “bare” supporting particle proceeds in much the same way as many smaller spherical particles bound to a sphere. In particular, we calculate *δR*_rh_ for ellipsoidal grafted particles by sequentially positioning *N* such particles randomly on a sphere and calculating *R*_rh_ of the grafted NP system after each particle is added. We may model a grafted polymer layer in a crude coarse-grained approximation by taking the ellipsoids to be prolate in form and oriented with their long axis of dimensions oriented normal to the supporting surface at the point of the ellipsoid attachment. Flexible polymers in solution are known to exhibit an ellipsoidal average shape in with appreciable anisotropy^90,91^ and fluctuations in their anisotropy have also been observed.^92^ We can expect the repulsive interchain interactions in the grafted layer to only enhance this tendency, which is the basic concept behind the “brush” picture of such grafted layers.^76,77^ In general, the shape anisotropy depends on polymer topology, chain stiffness, and polymer excluded volume interactions.^30,49,64,93,94^

**Fig. 12:**
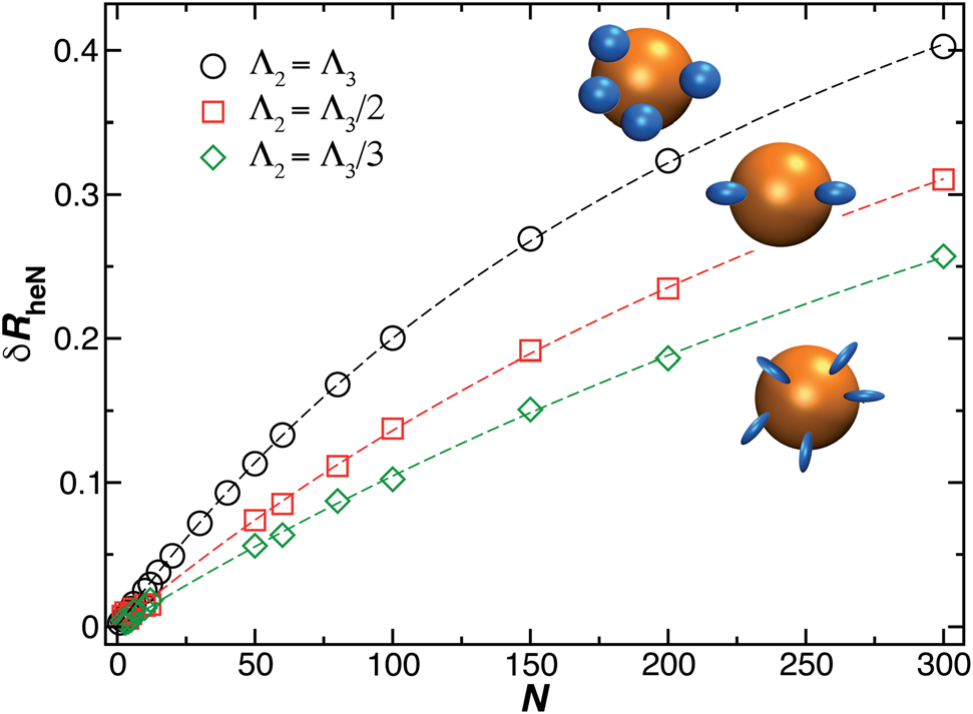
Change in the hydrodynamic radius for a spherical particle (gold sphere) due to the addition of *N* small ellipsoidal objects (blue particles). We find the data is well described by eqn (9) (dashed lines, with *ρ* ≥ 0.99).

The model of ellipsoidal chains grafted to a spherical particle in a perpendicular orientation is intended to describe grafted chains that do not have a strongly attractive interaction with the surface, and in the case of the presence of such attractive interactions, *e.g.*, when a polymer becomes bound to the substrate, we can simply modify the model so that the length of the ellipsoid of revolution along its symmetry axis (quantified by the largest eigenvalue of the radius of gyration tensor of the ellipsoid, *Λ*_3_) is made smaller than in the transverse direction (quantified by the smallest eigenvalue of the radius of gyration tensor of the ellipsoid, *Λ*_3_), *i.e.*, the bound polymer is then modeled by an oblate ellipsoid of revolution. Fig. 12 shows the results of our method, as just described for *δR*_rh_, where we model the ellipsoids as being prolate and oriented normal to the surface of the spherical particle surface. This is the most relevant case for modeling DNA molecules grafted to Au NPs. We see that eqn (9) describes our estimated values of *δR*_rh_ very well, even when the axial ratio of the ellipsoid is varied over a large range.

We should mention that many NPs form “auto-grafted” layers when exposed to a solution environment containing proteins and other biological macromolecules that “largely defines the biological identity of the particles”.^95,96^ There has been tremendous and ever-growing interest in the formation of this type of “corona” layer in connection with the targeting of NPs to various tissues in a medical science context^97^ and in understanding of toxicity of NPs in some types of NPs, which can be greatly affected by the nature of this interfacial NP coating.^98,99^ The nature of the interactions governing the formation of these layers can be subtle, and even NP size,^74^ other solution conditions being equal, can greatly alter the strength of the NP interaction with the binding proteins. It is this type of grafted polymer layer that is of greatest interest in medical and environmental impact studies,^100,101^ and thus in understanding and modeling the ultimate biological activity of NPs released into the environment or introduced into the body.^102,103^ There has also been a great and ongoing efforts aimed at the difficult problem of quantifying the interactions between NPs and proteins.^97^ It is further notable that the hydrodynamic size, *i.e.*, *R*_h_, of NPs is strongly implicated in their cellular uptake.^104^

### 3.5 Applications of particle with randomly attached smaller particles

The model of a spherical particle with smaller particles attached at random to its surface has been widely utilized as a basic model of the formation of protein layers on NPs, where this modeling is combined with dynamic light scattering modeling of *R*_h_ of the NP with bound proteins and thermodynamic modeling of the number of “bound” polymers based on simple Langmuir adsorption theory, extended to account for the cooperativity of binding (Hill model).^60,105–108^ Cooperativity in molecule binding that naturally arises from many internal degrees of freedom and multifunctionality of protein binding to interfaces.^109,110^ In particular, the average number of “bound” particles *N* is normally modeled as^60,105–108^10
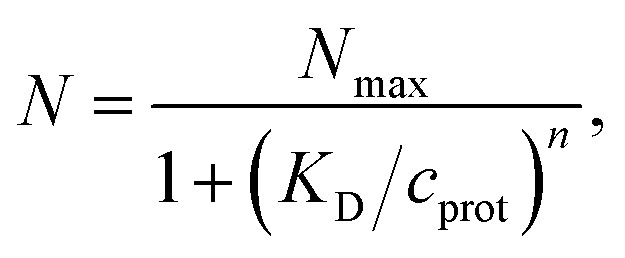
where *N*_max_ is the maximum number of proteins that will “fit” on the NP surface, defining “saturation coverage”, *c*_prot_ is the molar protein concentration in solution, *K*_D_ is the Langmuir dissociation constant, and *n* is the empirical Hill parameter quantifying the cooperativity of the assumed equilibrium molecular binding process. This type of Langmuir adsorption process has also been applied successfully to describe the equilibrium coverage and binding kinetics of inorganic NPs to macroscopic substrates^79^ and the kinetics of non-equilibrium irreversible binding of NPs to macroscopic interfaces. It should be possible to extend this type of modeling to describe the kinetics of protein binding to NPs^111^ under both equilibrium and non-equilibrium conditions to describe the growth of the protein corona layer or more generally the growth of layers on DNA ad synthetic polymer grafted layers based on methods, models and observations made before for polymers grafted and bound to plane surfaces.^68,69,112^

We emphasize that previous studies of *R*_h_ of proteins bound to their surfaces^60,105–108^ have completely neglected the role of hydrodynamic interactions on the *R*_h_ of NPs with bound proteins, but the analytic modeling of *R*_h_ has rather involved a simple interpolation relation between the limits of no attached proteins (the “bare” NP) and uniform coated sphere model as an approximate description of the high protein coverage limit. The utilization of eqn (9) above should allow a physically more faithful description of the hydrodynamic properties of this important class of naturally occurring grafted NPs. We finally note that the ZENO program allows the treatment of arbitrarily shaped NPs and the “grafting” of particles having general shape and orientation to these particles, based on modeling of the attachment process of these particles. Vargas-Lara *et al.*^94^ has discussed the calculation of the and intrinsic viscosity of particles and polymers having general shape, illustrating the application of ZENO in this generalized mode. We next turn to the modeling of many grafted polymer chains to a NP surface.

### 3.6 Spherical nanoparticle with many grafted small chains

**Fig. 13:**
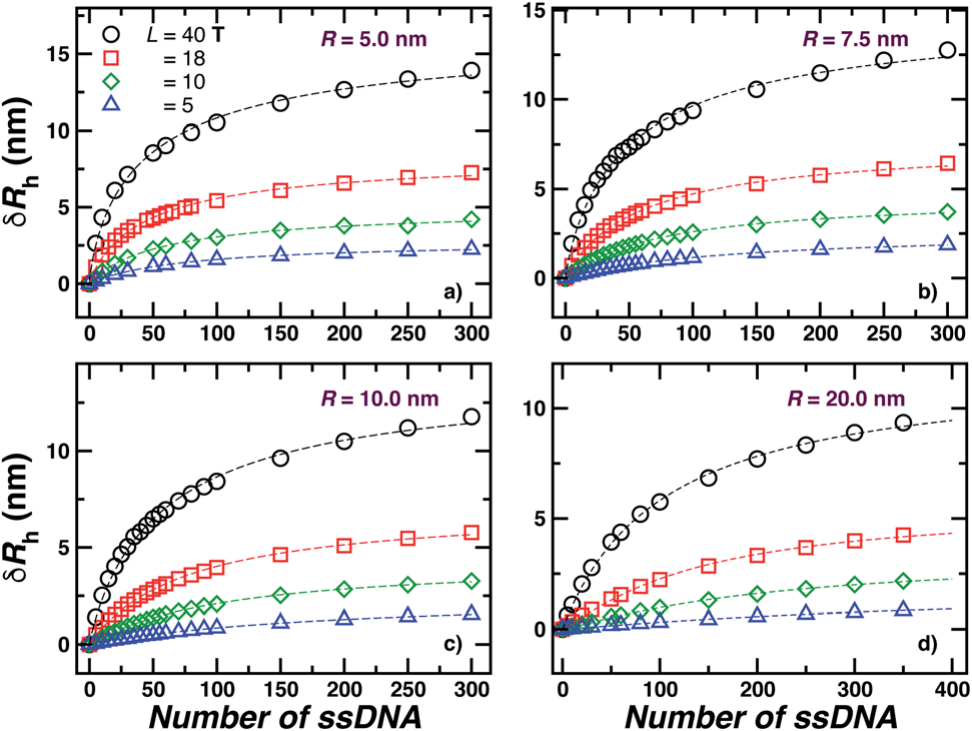
Comparison between all simulated systems (open symbols) and fits to eqn (11) dashed lines (*ρ* = 0.98). We find good agreement between eqn (11) and calculation results.

We finally consider the case in which the spherical NP is “decorated” with *N*_c_ ssDNA strands where each ssDNA strand has a fixed length *L*. For this case we find that eqn (9) must be modified to account for the polymeric nature of the grafted layer components. We find that for this case *δR*_h_ follows the relationship,11
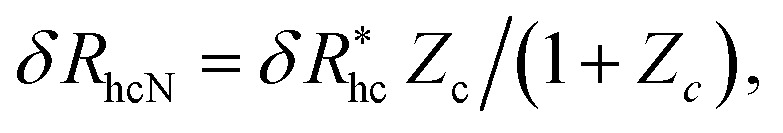
where 
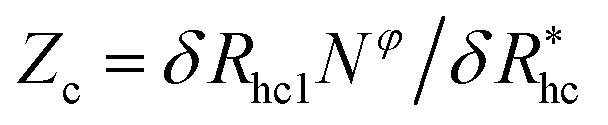
, where *δR*_hc1_ is enhancement of *R*_h_ due to a single chain and 
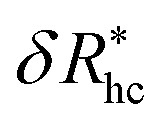
 is the saturation limit of the enhancement of *R*_h_ by the grafted layer (
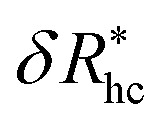
 is specified below). The “cross-over” exponent *φ* accounts for chain-core and chain–chain interactions, and we find below that this quantity empirically ranges from *φ* = 0.58 for grafted flexible polymers to *φ* = 0.58 for grafted spheres. Fig. 13 shows the simulation results for all systems considered in this study (particles interacting attractively) along with fits to eqn (11).

**Fig. 14:**
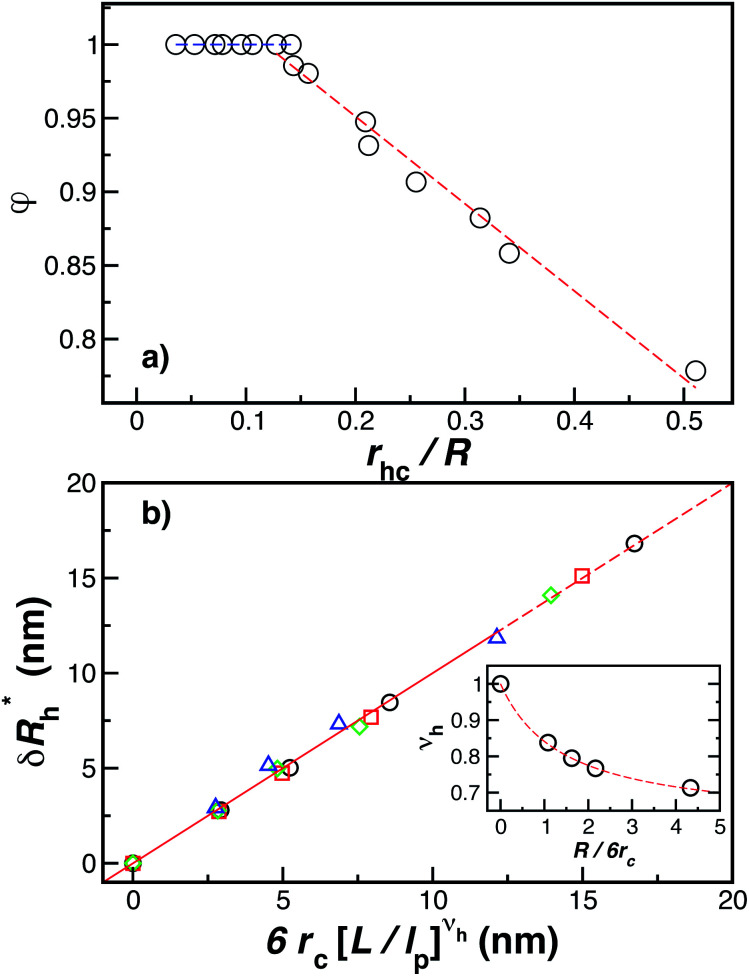
The parameters (a) *φ* and (b) 
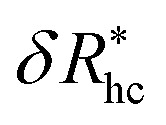
 as a function of the DNA-grafted structural parameters. The inset in panel (b) shows the *R* dependence of *ν*_rmh_. Dashed lines are guide for the eye.

Fig. 14(a) and (b) indicate that there are two coverage regimes, one for *r*_hc_/*R* ≤ 0.14 where the chain–core interaction is weak so that *φ* = 1, and a second regime, *r*_hc_/*R* > 0.14, where *φ* decreases nearly linearly with increasing *r*_hc_/*R*. Evidently, the curvature of the spherical core particle exerts an appreciable effect on the extent of the grafted layer contribution to *R*_h_. We also see that the saturation coverage 
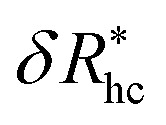
 follows a power law dependence on the ratio *L*/*l*_p_, where *l*_p_ is the polymer persistence length, and the effective power *ν*_h_ is a function of the ratio, *R*/*r*_c_. Here we follow a similar procedure to our previous study of DNA polymer chains under strong geometrical conditions between two interfaces^89^ in developing an approximant for the average thickness of the grafted layer, 
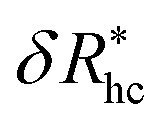
, and12
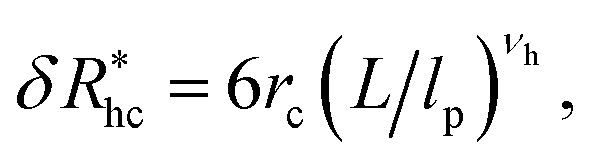

13 *ν*_h_ = [1 + 0.43(*R*/6*r*_c_)]/[1 + 0.70(*R*/6*r*_c_)].

**Fig. 15:**
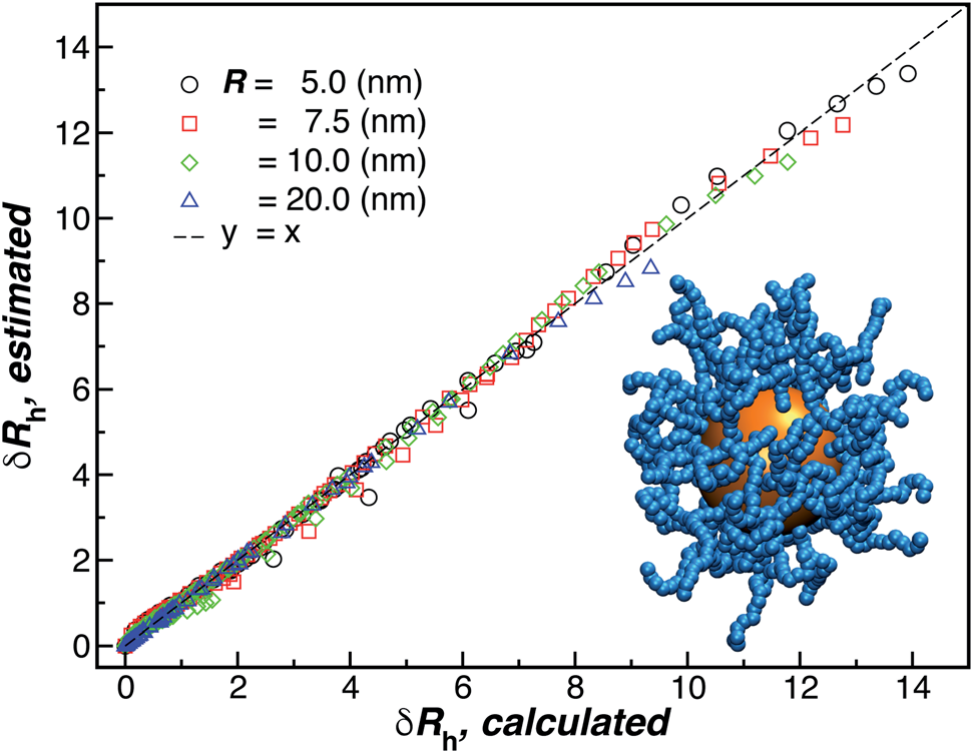
Comparison between *δR*_h_ values obtained by simulations *versus* the ones estimated by using eqn (11). The dashed line corresponds to a linear relation *y* = *x* with *ρ* = 0.98.

Finally, we show in Fig. 15 all our simulation observations of *R*_h_ of polymer-grafted NPs can be described by the universal scaling relation, *R*_h_ = *R*_h_(core) + *δR*_h_, where *δR*_h_ is defined by eqn (11). This expression can be recognized as rather similar to the Kirkwood–Risemann mean-field theory for *R*_h_ of a flexible chain, which again has the same cross-over form,^85,86,113,114^14
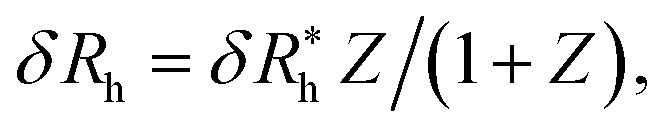
where 
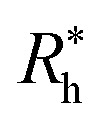
 is proportional to the chain *R*_g_ and the hydrodynamic interaction strength parameter *Z*. Here, 
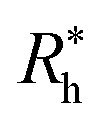
 is the limit of strong hydrodynamic interaction where the polymer domain becomes hydrodynamically “impermeable” where the hydrodynamic interaction strength *Z* scales as the number of chain segments *N* divided by *R*_g_^*d*−2^ where the *d* − 2 power reflects the dimensional scaling of capacity/hydrodynamic radius.^85^ A renormalization group (RG) calculation of *R*_h_ of flexible polymers also leads to eqn (11) as a leading expansion in the variable *ϕ*, which equals, *ϕ* = (4 − *d*)/2 ≡ *ε*/2, where *d* is the spatial dimensionality.^115,116^ The “cross-over” expression for *R*_h_ of flexible polymers as function of the hydrodynamic interaction and excluded volume interaction strength becomes more complicated in functional form beyond the leading order in the *ε*-expansion.^115,116^ Fortunately, variable hydrodynamic interactions fluctuations are normally less prevalent in polymer-grafted NPs having a moderate grafting density than isolated flexible polymer chains in solution, making the mean-field cross-over expansion, eqn (11), a rather good approximation.

## 4 Other nanoparticle solution properties relating to “size” and average shape

### 4.1 Ratio between the hydrodynamic radius and radius of gyration

Another important quantity that helps the characterization of these particles constitutes the ratio *R*_h_/*R*_g_, where *R*_g_ is the radius of gyration of the particle. In particular, for a spherical particle equals, 
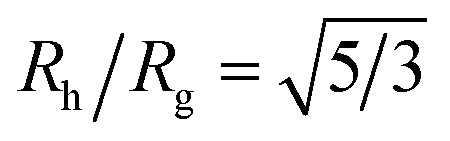
. Experimentally, this quantity can be obtained by X-ray and neutron scattering and depending on the technique utilized, and the contrast, *R*_h_/*R*_g_ for the DNA-grafted NP may vary. Fig. 17 shows *R*_h_/*R*_g_ calculated numerically considering the scatter particles having equal contrast than the nanoparticle core (red squares), or neutrons (blue triangles).

We find that the mean dimensions of the grafted chains are not significantly altered from their random coil conformation in solution even at saturation coverage, so that the term “brush” is potentially misleading. In particular, we find that an approximately linear increase of *R*_h_ with the length of the grafted chains at a saturation coverage cannot be taken to imply that the grafted chains adopt highly extended worm-like chain configurations as often suggested in cartoon models of grafted polymer layers. As one of the most important results of our paper, we have developed a general cross-over function describing *R*_h_ for NPs having variable core size, number of grafted chains, and the variable grafted chain length, based on a consideration of the chains as spheres or ellipsoids. We validated our results against numerous simulations for these structures, and against experiments under conditions where the interfacial grafting densities could be readily investigated. We expect these results will be useful in the characterization of surface-grafted NPs.

Our numerical calculations indicate that the fluctuations in the shape of the polymers in the grafted layer can give raise the appreciable fluctuations in *R*_h_ about its average values even when the NPs are monodispersed in NP core size, have the same number of grafted chains and the grafted chains all have the same length. With increasing grafting density, the variance of these fluctuations first increases and peaks and they decrease as the layer becomes fully saturated. We expect these estimates of the fluctuations in the shape of NPs to be important for understanding the interactions between the NPs and their interactions with biological molecules and structures.

The quantification of the influence of the interaction of the grafted chain and the surface of the NP core is also a novel aspect of the present work. We find that this interaction can greatly influence the effective NP size (*R*_h_), but in the case of Au NPs having a size truly in the nanometer scale, *i.e.*, 5 nm, this effect is apparently small because of the weakness of the ssDNA–Au NP interaction for particles in this size range and for the solution conditions investigated.

Since the development of a saturated grafted layer is expected to be crucial in many applications in which interfacial stabilization of NPs is required, it is important to develop new experimental methods to quantify this aspect of polymer-grafted NPs. We suggest that this saturation effect, and more generally, the nature of the polymer grafted layer, can be quantified through a combination of static and dynamic light scattering, neutron, and X-ray scattering and solution viscosity measurements where the emphasis is on measured routinely properties by each of these methods.

As described in the Introduction, the presence of a grafted layer of polymer chains metal and other dense NP cores can lead large values of the ratio *R*_h_/*R*_g_ that are significantly larger than this ratio for spheres so that the measurement of these solution properties can provide very useful information about the NP density profile, especially in the case of NPs having a nearly spherical shape (particle shape anisotropy also impacts *R*_h_/*R*_g_, usually making this ratio take on smaller rather than larger values than the uniform sphere^64^). Although the qualitative origin of these large values has long been understood, it has been difficult to use this effect to obtain quantitative information about the NP structure because of the absence of an analytic theory for calculating *R*_h_ of polymer grafted NPs and other microgel particles having diffuse interfaces.

The computational scheme for polymer grafted NPs using ZENO in conjunction with molecular dynamics simulations, allows this type of quantitative analysis of the physical significance of anomalously large values of *R*_h_/*R*_g_. We first illustrate the influence of particle anisotropy in the case of a dumbbell configuration of a sphere attached to another sphere whose size is generally different. Wyatt has recently provided an estimate of *R*_g_^2^ of this type of dumbbell of spherical particles,^117^15
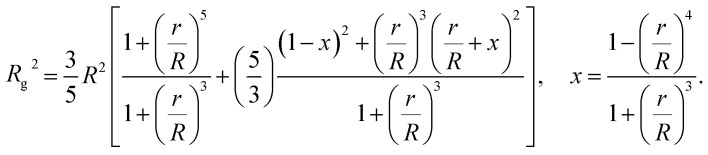


**Fig. 16:**
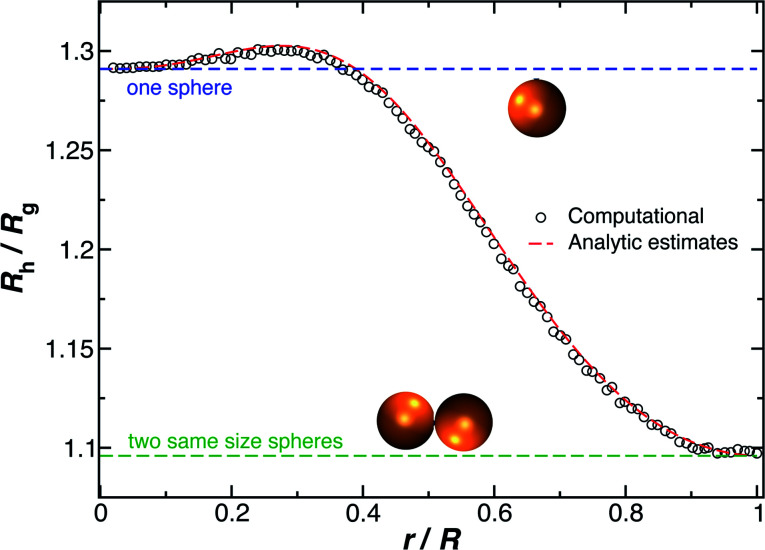
The hydrodynamic radius *R*_h_ normalized by the radius of gyration *R*_g_ for a dumbbell as a function of the ratio (*r*/*R*). The solid symbols are calculations and the analytic results (dashed lines with *ρ* = 0.98) are obtained by combining eqn (6) with eqn (15).

We combine this result with eqn (5) to estimate *R*_h_/*R*_g_ for the dumbbell as a function of the ratio (*r*/*R*) and show the calculation in Fig. 16. We also include the analytic calculations for the particular cases including and individual sphere, 
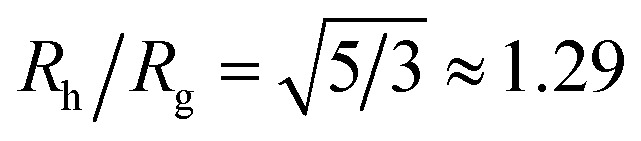
 and for two touching spheres having the same radii, 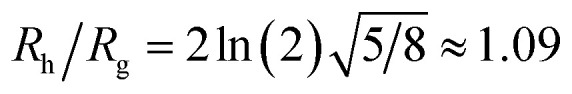. We see that increasing the radius of the grafted sphere, and thus anisotropy of the dumbbell, causes a decrease in *R*_h_/*R*_g_, although *R*_h_/*R*_g_ exhibits an unexpected shallow maximum when the attached sphere has a radius of about 0.3 times the radius of the “core” particle. This overshooting of the hard sphere value of the ratio *R*_h_/*R*_g_ has been observed in simulations of high generation dendrimers under good solvent conditions where the strong repulsive interchain excluded volume interactions within these densely branched molecules cause leads to formation of distinct domains within the molecule and to an associated “crenulated” surface morphology.^38^ The most probable configurations of linear and branched polymer chains under equilibrium conditions are rather anisotropic, and corresponding *R*_h_/*R*_g_ is normally significantly less than 1.^64^ The density profile of the polymer or particle can also impact this ratio and measurements of *R*_h_ and *R*_g_ of polymer grafted NPs are instructive in illustrating this effect, as we now describe. There are some complications with such measurements that need to be described first, however.

**Fig. 17:**
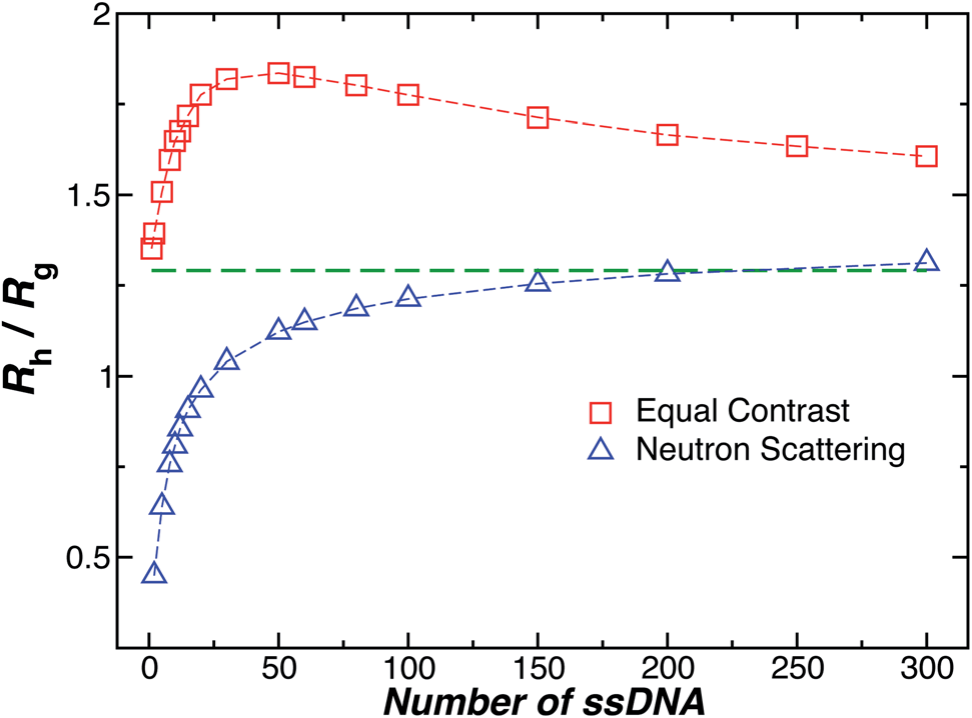
The hydrodynamic radius *R*_h_ normalized by the radius of gyration *R*_g_ for a 5 nm in radius NP decorated with different number of strands having each strands 18 **T** bases. Dashed lines guide the eye.

Although a combination of static and dynamic scattering measurements can be highly revealing about the nature of the grafted polymer layer on NPs, it must be remembered that scattering measurements often depend on scattering contrast, which can complicate this type of analysis. For example, the estimation of *R*_g_ by X-rays is normally dominated by the scattering of the metal core of the NP, making the grafted layer nearly “invisible”. By deuterating the polymer chains, the neutron scattering of this type of particles can be dominated by the grafted chains and the metal nanoparticle can be made to be effectively “invisible” by appropriate contrast matching. For nanoparticles composed of the same chemical species, the entire particle has essentially the same contrast, which is another type of “soft” NP commonly encountered in applications. Fig. 17 shows *R*_h_/*R*_g_ based on these different experimental methods. In this figure, the black line shows the case where all contrast comes from the NP core and the blue line indicates an ideal neutron measurement estimate of *R*_h_/*R*_g_ where all scattering contrast for *R*_g_ is derived from the polymer grafted chains. The uniform scattering case, appropriate for NPs having a uniform chemistry, is shown as the red curve in Fig. 17. The ratio *R*_h_/*R*_g_ rises with surfaces coverage and peaks near the saturation coverage ≈50 grafted strands for this NP. The neutron estimates of *R*_g_, in combination with *R*_h_ measurements, also seem to provide an effective methodology for identification of the formation of a “saturated” grafted layer. Note that *R*_h_/*R*_g_ significantly exceeds the value for a spherical particle, *R*_h_/*R*_g_ = 1.29 for the uniform contrast case, where the excess value provides valuable information about the diffuse grafted polymer layer. Values of *R*_h_/*R*_g_ corresponding to the equal contrast estimates in Fig. 17 have often been reported in microgel NPs.^32,33^ Values of *R*_h_/*R*_g_ significantly larger than the hard sphere value have also been observed polyelectrolyte complexes of DNA and polyethyleneamine^41^ of interest in gene therapy and antiviral medicines.^118^ In these systems, the DNA forms a dense core structure analogous to the metal nanoparticle in the present paper and the polyethylene molecules form a diffuse peripheral layer structurally analogous to the grafted ssDNA layer. We may also the presence of the spike proteins on COVID virus and other spherical viruses with spike like structures on their surfaces to lead to especially large values of *R*_h_/*R*_g_. We find that this ratio can become as large as *R*_h_/*R*_g_ = 2.14, when the grafted polymers are assumed to adopt a rod-like conformation when *N* is large. Hydrodynamically, these particles behave as if they are much larger than their spherical core, an effect that we term the “dandelion effect”.

### 4.2 Intrinsic viscosity

**Fig. 18:**
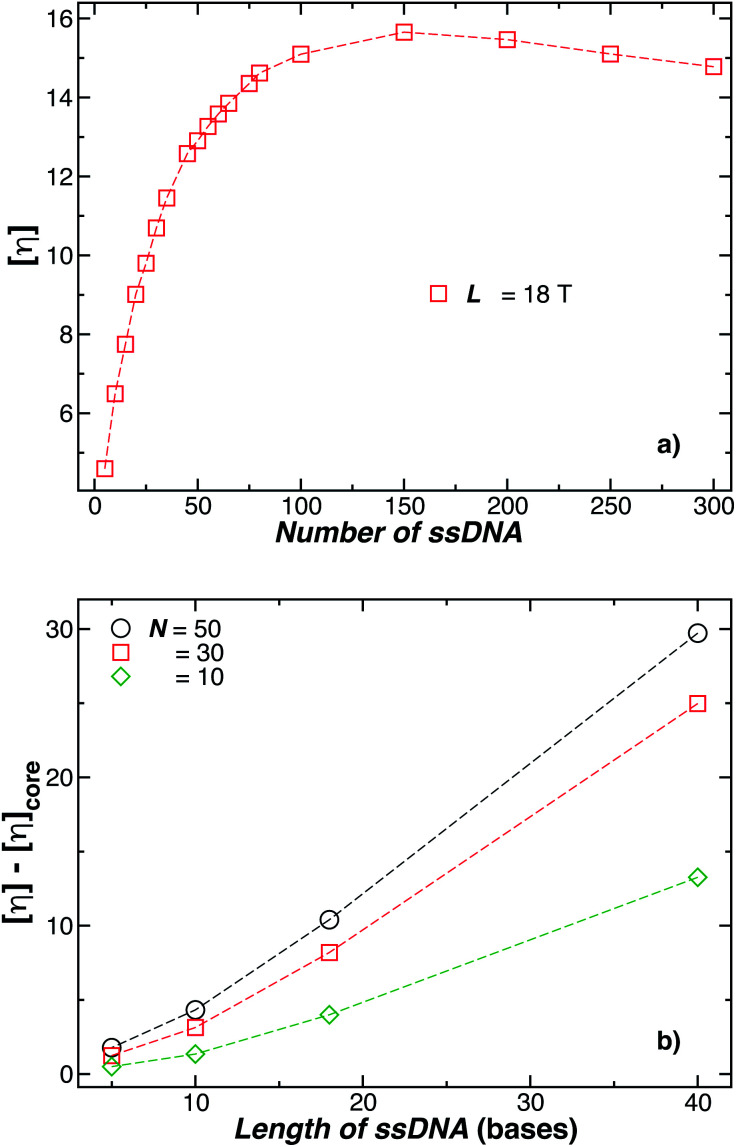
In (a) the intrinsic viscosity [*η*] for a 5 nm in radius NP decorated with different number of strands having each strand 18 **T** bases. Evidently, [*η*] reaches its maximum value when *N* ≈ 150 chains, but [*η*] must approach the Einstein value 5/2 in the dense “brush limit” when the grafting density becomes uniform. (b) Enhancement of [*η*] for the polymer-grafted NPs as a function of the chain length. The number of chains attached on each NP is reported in the legend. Dashed lines guide the eye.

Although *R*_h_ and *R*_g_ are the primary measures of NP size utilized in their solution characterization, [*η*] and second osmotic virial coefficient *A*_2_ are often utilized to characterize polymers in solution^119,120^ and we may expect these solution properties to also become important in the characterization of NPs in the future. Here, [*η*] is defined as,16
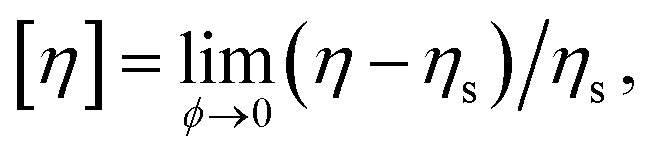
where *η* and *η*_s_ are the solution and solvent viscosity, respectively, and *ϕ* is the NP volume fraction and correspondingly *A*_2_ is the leading term in the solution osmotic pressure as a function of *ϕ*. Although a simple solution property in principle, there is no general-purpose program that allows for the calculation of *A*_2_ for polymer grafted NPs, but the program ZENO currently allows for the computation of [*η*] with no greater effort than *R*_h_, where again the uncertainty estimate from value from hydrodynamics has been estimated to be about 1%.^43^ As an illustration of this type computation, we show in Fig. 18(a) estimates of [*η*] of DNA grafted Au NPs with a core radius of 5 nm based on the same grafted NP model considered above in our *R*_h_ computations. In Fig. 18(a) we find that [*η*] saturates when the numbers of grafted chains are approximately 100, each chain is 18 **T** bases in length. We should then also be able to estimate the grafting saturation condition from dilute solution viscosity measurements. Fig. 18(b) shows that we may also gain insight into the extent of the grafted layer by taking the difference, *δ*[*η*] = [*η*](NP) − [*η*](sphere), between [*η*] of the polymer-grafted NP and the NP core, where *η* for an spherical particle equals, [*η*] = 2.5,^121^ Δ[*η*] then initially increases with the length of the chains and the number of grafted polymers, but this effect ultimately saturates. However, at very high grafting density the grafted chain segmental density must become uniform and thus ultimately [*η*] decreases to the value 2.5 for a sphere in this “dense brush” limit.

It is evident that the common procedure,^122–125^ of determining the “effective volume fraction” *ϕ* of suspensions by matching the linear concentration dependence of the solution viscosity to the Einstein result, [*η*] = 2.5 can be problematic when the particles have diffuse interfacial layers, as often found for grafted NPs and “soft” NPs. It is then quite understandable why the apparent volume fraction is often reported to be much larger than 1.^125^ We suggest this procedure of volume fraction estimation should be avoided and solution properties based on a dubious “mapping” onto hard sphere suspensions should be replaced using the reduced concentration variable, [*η*]*ϕ*.

## 5 Conclusions

We have developed series of models of increasing molecular faithfulness of individual polymer chains grafted onto a spherical nanoparticle (NP) structure (spheres, ellipsoids, semi-flexible polymer chains with excluded volume interactions), and we then extended the model to describe collections of these coarse-grained model polymers using a combination of molecular dynamics simulations of the conformational structure of the grafted polymer layers and the path-integration program ZENO^43^ to calculate basic solution hydrodynamic properties, hydrodynamic radius (*R*_h_), radius of gyration (*R*_g_), and intrinsic viscosity ([*η*]). We particularly emphasize *R*_h_ calculations because these measurements have been found to be especially useful in applications aimed at characterizing these NPs in relation to biophysical activity in many emerging applications using this type of NP for drug delivery, diagnostics, and other medical applications, and in connection with assessing the toxicity of NPs released into the environment, as summarized in our introduction. The potential applications are made broader by the fact that when NPs are released into the environment, they tend to spontaneously acquire a grafted layer or “corona” layer on their surfaces (auto-grafting) arising from the physical binding of polymers in their environment to the NPs and our modeling methodology can be used also for modeling such systems under thermodynamic conditions where the molecular binding to the “bare” NPs can be described by a reversible reaction process.

We have found that the presence of the grafted layer can greatly alter the “effective size” of the inorganic NPs to which they are grafted, even when the polymer grafting density is relatively low. Moreover, the average hydrodynamic thickness, of this grafted layer, and fluctuations in its effective thickness and correspondingly the NP hydrodynamic size, are also highly dependent on the length of the grafted chains and grafting density, the strength of the interaction potential between the polymers and the surface of the core NP, and the size of the NP core relative to the radius of gyration of the grafted polymer chains. The strong hydrodynamic screening effect^88^ within the diffuse grafted polymer layer then allows us to understand the relatively large values (significantly larger that the ratio of hard sphere particles) of the ratio *R*_h_/*R*_g_ observed in polymer grafted NPs, and other soft gel particle systems such microgel particles. Intrinsic viscosity derived measures of hydrodynamic size can be likewise expected to be “anomalously large”, but we do not emphasize these measurements because of their more limited use in NP characterization.

Our results for the solution size ratio *R*_h_/*R*_g_ of polymer grafted NPs are contrasted with those for isolated linear polymers, and even polymers having regular branched architectures such as star and ring polymers, where *R*_h_/*R*_g_ is generally less than the value for hard spheres, often taking significantly smaller values^30,50^ The extent of this hydrodynamic “screening effect”^88^ depends on the polymer grafting density and polymer chain length, and we also investigate the distribution and variance of these solution properties that arise from fluctuations in the conformation of the grafted chains, as well as the positions of grafting on the supporting sphere. Fluctuations in the grafted layer structure can be expected to alter the interaction between these complex NPs and with biomacromolecules of relevance to their medical applications.

We then expect this computational methodology, in conjunction of experimental measurements, should be useful for characterizing polymer-grafted NPs for use in many applications in material science and nanomedicine and in needed studies aimed at evaluating the environmental impact of NPs in the environment. Although we have combined our applications to spherical core particles with grafted single stranded DNA layers, the ZENO methodology, in conjunction with molecular dynamics simulation, allows for the treatment of NPs having essentially arbitrary shape and polymer monomer structure and having variable molecular rigidity and local intermolecular potential interactions. Previous attempts at describing polymer grafted NPs have generally been based on heuristic “layer models” that do not consider hydrodynamic interaction effects arising between the molecules in the grafted layer and the NP to which they are grafted or between the chains in the layer nor the effect of fluctuations in the overall thickness and shape of the grafted layer that arise in conformational fluctuations of the polymer chains in the layer or chain conformational fluctuations. In contrast, our computational method, should enable the quantification of these fluctuation effects through appropriately designed simulations and measurements.

As a final point, we emphasize that the present work has emphasized the characterization of individual DNA grafted Au nanoparticles based on solution properties performed at high dilution such as the hydrodynamic radius and intrinsic viscosity. Many applications of polymer grafted nanoparticles, on the other hand, involve higher NP concentrations where such nanoparticles start clustering, leading to evident changes in the optical properties of these solutions^15,126,127^ that are useful in colorimetric sensing applications, *etc.* The effects of nanoparticle association, such as dimer formation,^128–130^ is clearly discernible experimentally. Dimerization of DNA grafted nanoparticles has been modeled by the same coarse-grained model as utilized in the present paper^26,27,46^ and it would be a relatively simple matter to calculate the size distribution of the hydrodynamic radius of these dimers under different conditions to compare with dynamic light scattering and high resolution sedimentation coefficient measurements of these dimers based on simulations of the kind described in the present paper. Very interesting measurements have been performed showing the change in the hydrodynamic radius of DNA grafted nanoparticles when complementary linear chains are physically bound to the grafted chains.^131,132^ Under more general circumstances, the binding of molecules on drug end-functionalized grafted polymers on nanoparticles could be modeled to determine the degree of this binding to the grafted nanoparticle. This type of information would obviously be useful in many biomedical applications. There has also been great interest in creating and controlling larger clusters of DNA grafted nanoparticles and even crystalline arrays of nanoparticles.^25,29,133–136^ Sheets or “membranes” of DNA grafted nanoparticles have also been synthesized.^137^ There is thus considerable scope for extending the present to seemingly endless configurations of DNA grafted nanoparticle that can be created with these inherently modular materials.

## Conflicts of interest

We report no known conflict of interest.

## Supplementary Material
